# Pain assessment and management in care homes: understanding the context through a scoping review

**DOI:** 10.1186/s12877-021-02333-4

**Published:** 2021-07-18

**Authors:** Jan Pringle, Ana Sofia Alvarado Vázquez Mellado, Erna Haraldsdottir, Fiona Kelly, Jo Hockley

**Affiliations:** 1 Scottish Collaboration for Public Health Research and Policy, University of Edinburgh, Edinburgh, UK; 2 St Columbas Hospice and Queen Margaret University, Edinburgh, UK; 3 School of Health Sciences, Queen Margaret University, Edinburgh, East Lothian UK; 4 Usher Institute, University of Edinburgh, Edinburgh, UK

**Keywords:** Pain assessment, Pain management, Care homes, Scoping review

## Abstract

**Background:**

Internationally, 2–5% of people live in residential or nursing homes, many with multi-morbidities, including severe cognitive impairment. Pain is frequently considered an expected part of old age and morbidity, and may often be either under-reported by care home residents, or go unrecognized by care staff. We conducted a systematic scoping review to explore the complexity of pain recognition, assessment and treatment for residents living in care homes, and to understand the contexts that might influence its management.

**Methods:**

Scoping review using the methodological framework of Levac and colleagues. Articles were included if they examined pain assessment and/or management, for care or nursing home residents. We searched Medline, CINAHL, ASSIA, PsycINFO, EMBASE, Cochrane Library, and Google Scholar; reference lists were also screened, and website searches carried out of key organisations. Conversations with 16 local care home managers were included to gain an understanding of their perspective.

**Results:**

Inclusion criteria were met by 109 studies. Three overarching themes were identified: *Staff factors and beliefs* - in relation to pain assessment and management (e.g. experience, qualifications) and beliefs and perceptions relating to pain. *Pain assessment* – including use of pain assessment tools and assessment/management for residents with cognitive impairment. *Interventions* - including efficacy/effects (pharmaceutical/non pharmaceutical), and pain training interventions and their outcomes.

Overall findings from the review indicated a lack of training and staff confidence in relation to pain assessment and management. This was particularly the case for residents with dementia.

**Conclusions:**

Further training and detailed guidelines for the appropriate assessment and treatment of pain are required by care home staff. Professionals external to the care home environment need to be aware of the issues facing care homes staff and residents in order to target their input in the most appropriate way.

**Supplementary Information:**

The online version contains supplementary material available at 10.1186/s12877-021-02333-4.

## Background

Internationally, 2–5% of people live in residential or nursing care homes, with almost 60% of residents being over 85 years of age [1]; many live with multi-morbidities, including severe cognitive impairment [2]. Pain is frequently considered an expected part of old age and morbidity, and as a consequence is often under-reported by care home residents, or may go unrecognized by care staff [3].

The International Association for the Study of Pain (IASP) [4] estimates that 20% of the adult population are living with chronic pain. They consider pain to be individual and subjective, and assert that the inability to communicate pain verbally, for example in people with dementia, does not negate the possibility of pain being present, and the need for treatment [4].

Where the presence of pain cannot be verbalized, pain behaviors may include guarding, agitation, facial expression, or altered mobility [4]. However, it is unclear how well such factors are understood and, when pain is identified as the probable cause of such behaviour, whether this is acted upon by care home staff [5]. In addition, where pain is recognized and treated, the IASP advises caution with older people, who are generally less tolerant of analgesia, and may experience side effects such as sedation or confusion if the analgesia type and dose are not chosen with care [4]. In addition, over or misprescribing may lead to drug related problems for nursing home residents [6]. Non-pharmacological treatments or adjuncts are therefore worth considering for treatment of pain in older age. In addition to such concerns, the influence of emotional state on pain is a significant factor, which is not always acknowledged [7].

While appropriate assessment of pain is an essential step to effective treatment [8], and various pain assessment tools have been developed [9], pain assessment and management in care homes is complex. To further understand these complexities, we undertook a scoping review to explore the contexts which may influence pain assessment and management for care home residents.

## Methods

### Review category

This scoping review was carried out using the methodological framework of Levac et al. [10] involving six stages noted in Table 1.

**Table 1:** Methodological framework (Levac et al., 2010)

Stage	Actions undertaken
Stage 1	Clarifying and linking the purpose and research question
Stage 2	Balancing feasibility with breadth and comprehensiveness of the scoping process
Stage 3	Using an iterative team approach to selecting studies
Stage 4	Extracting data
Stage 5	Incorporating a numerical summary and qualitative thematic analysis when reporting results (and considering implications for policy, practice, research)
Stage 6	Consultation with stakeholders as a knowledge translation component

### Review aim

The aim of the review was to summarize a range of evidence relating to the assessment and management of different types of pain experienced by frail older people living in care homes. Stakeholder opinions (care home staff/managers) were gathered to further inform evidence synthesis (Stage 6: Levac et al., 2010). Informal, face-to-face conversations with care home staff took place, to elicit their views and knowledge about pain assessment and management. These meetings were carried out as an adjunct to the scoping review (to fulfil stage 6, as indicated previously), to ascertain if local staff views shared similarities with the findings of the review, or displayed differences. The review was conducted between September 2019 and March 2020.

### Search strategy and selection criteria

#### Key words/MeSH terms

Key words relating to pain (e.g. pain, soreness, discomfort, allodynia, neuralgia, neuritis, neuropathy, sensitivity, dysesthesia, hyperalgesia etc.), and pain assessment or management, were combined with care home terminology (residential, care, nursing, residents, patients etc.) to acknowledge the multiple terms that may be used to describe this type of care setting. Terms were combined using Boolean AND/OR strategies, to ensure the most relevant work was accessed (e.g. pain OR discomfort OR neuralgia AND care home OR residential care OR nursing home etc), as detailed in the search strategy developed by the research team (Additional file 1). Due to time and resource limitations, papers not available or translated into English language were excluded

#### Types of study method

All study methods, and grey literature/reports were open for inclusion; the review aimed to give a broad perspective on the topic area, rather than an in-depth analysis from a narrower viewpoint. Theses and dissertations were excluded, unless summarised into shorter papers. This approach helped to balance the need for feasibility, with breadth and comprehensiveness (Levac et al., 2010: Stage 2).

#### Databases

Databases searched included: Medline, CINAHL, ASSIA, PsycINFO, EMBASE, Cochrane Library, and Google Scholar. Reference lists of relevant items were screened, and website searches carried out for key organisation reports (e.g. Age UK, The Care Quality Commission, National Care Homes Association, International Association for the Study of Pain, The British Pain Society etc.) to ensure a wide range of relevant literature was accessed.

### Inclusion and exclusion criteria are summarised in Table 2

#### Screening and selection methods

**Fig. 1:**
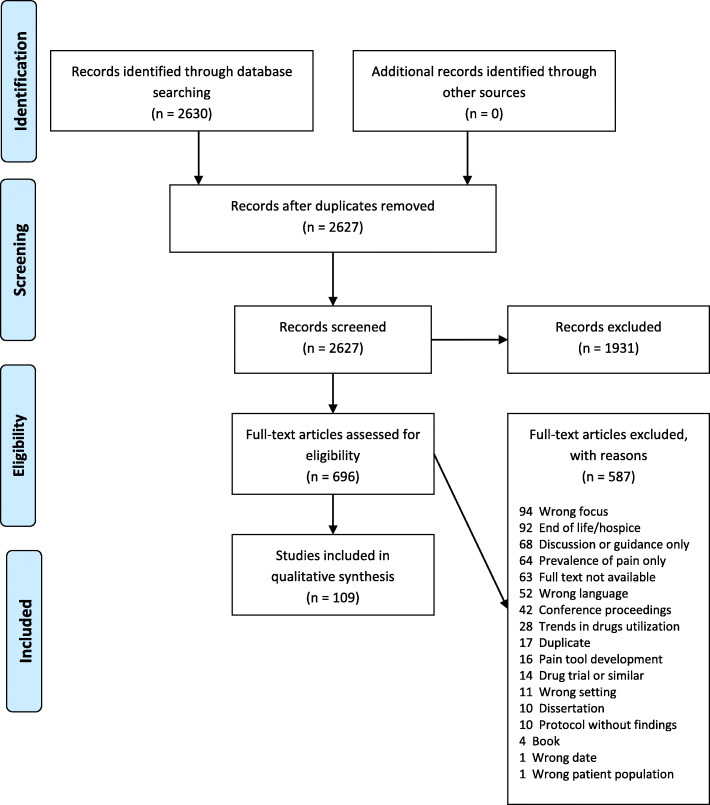
PRISMA diagram

Two reviewers (JP & SA) blind screened papers independently at all stages of the screening process, with disagreements being resolved by further discussion. A PRISMA flow diagram was prepared detailing the selection process – see Fig. 1.

**Table 2:** Inclusion and exclusion criteria

Inclusion criteria	Exclusion criteria
• Residential or nursing homes • Adults living in the above accommodation type • All types/methods of research or publication, including grey literature, apart from dissertations/theses • Studies relating to pain assessment and/or management • Outcomes relating to pain management effectiveness (e.g. pain level alteration, QoL, well-being outcomes, mood, behaviour, barriers or facilitators etc.) • Any geographical location • Publication within last 10 years (to relevance to current practice)	• People living at home, in hostels (with minimal supervision or care), in sheltered housing, hospices, or under long stay hospital care • Dissertations/theses (due to typical length, and resource limitations) • Publications or reports where an English language translation is not available • Studies that do not focus on pain assessment or management (e.g. prevalence of pain only) • Outcomes that do not relate to pain management • Studies or reports without outcomes (e.g. protocols) • Studies examining pain assessment tool development or validity as sole focus

QoL = quality of life

#### Data extraction and quality assessment

Data from individual studies or reports were extracted onto a pre-designed table, and cross-checked by two reviewers (JP & SA). We did not aim to produce a detailed quality appraisal of included studies or exclude studies on the grounds of quality, as per scoping review guidelines [11], but noted any quality limitations to further inform the review.

#### Analysis methods

We conducted a thematic analysis of the papers reviewed, and an analysis of stakeholder findings from 16 care homes. We sought to identify commonalities across studies and group these into areas of shared interest. To assist in this process, we used mind mapping [12, 13], in order to interpret and display associations between individual findings, and to identify common themes across studies. This approach was chosen because, with regard to analysing and improving health provision, there often needs to be “quite specific feedback about what works, where improvements can be made and what barriers there may be to accessing services. This type of feedback can be clearly represented in a mind map” [13].

Two researchers (JP, ASAVM) carried out an initial grouping, which was then discussed and amended with other team members, until agreement was reached. In particular, all data were examined to identify organisational factors, such as internal and external barriers or facilitators that might either help or hinder pain assessment and management. Links and implications for policy, practice and research were sought within the analysis process (Levac et al., 2010: Stage 5).

## Results

### Overview

One hundred nine studies were found that fulfilled all inclusion criteria. An overview of these studies is presented in Additional file 2. Due to the large number of studies, each tabulated ‘included study’ (IncS) was allocated a number (e.g. IncS 1, 2 etc) for identification within the following text. Full references details for included studies are presented below the main reference list.

There was a diverse geographical spread across 17 countries, indicating global interest and potential concern about the issues surrounding pain assessment and management in care homes.

### Methods and appraisal of included studies

Research methods used were diverse, including 13 RCTs, 18 non-randomised trials (typically pre/post-test studies), 16 qualitative studies, 30 of cross-sectional design, and 13 systematic reviews. Where studies were longitudinal (mainly RCTs) the longest duration of follow up was 6 months. While the majority of studies clearly detailed the methods used, study limitations largely related to methodological issues (e.g. cross sectional design) or small participant numbers. The difficulties inherent in including care home participants in reseach, who by the nature of their situation are vulnerable, was not widely acknowledged.

### Analysis

Our analysis provided a breakdown of topics of interest across and between studies, and revealed six initial themes:

- Staff factors in relation to pain assessment and management (e.g. experience, qualifications etc.)
- Beliefs and perceptions relating to pain (staff, residents, relatives)
- Pain assessment tools
- Pain assessment and management for nursing care home residents with cognitive impairment
- Pain intervention efficacy/effects (pharmaceutical/non pharmaceutical)
- Pain training interventions and their outcomes

**Fig. 2:**
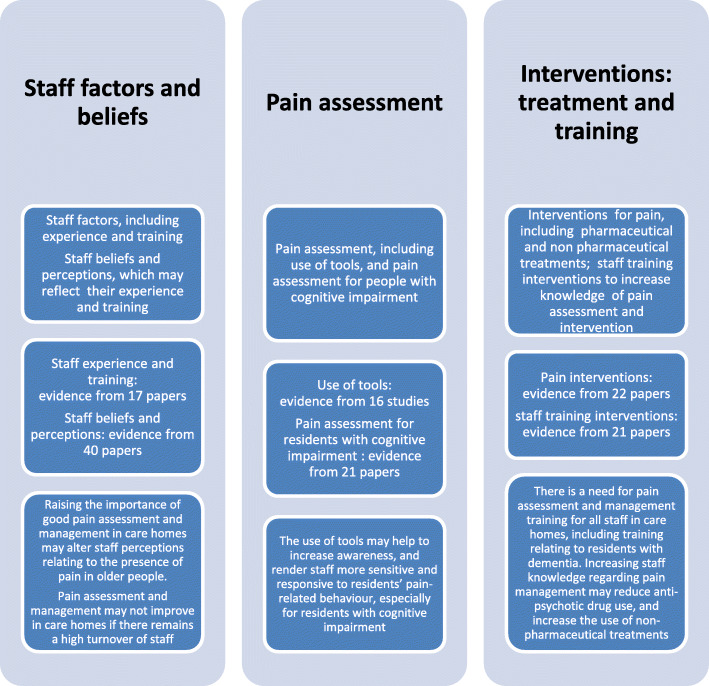
Summary of findings

These six themes were further amalgamated into three broader overarching themes or categories: *Staff factors and beliefs, Pain assessment, and Interventions*, as illustrated in Fig. 2. Due to some studies being multifaceted, results from a particular study may appear in more than one category.

### Staff factors and beliefs

There were a number of included papers (*n* = 17) that explored the training level and experience of health care staff, and the impact this had on pain recognition and practices relating to pain management.

For example, Takai et al. (IncS 94) reported a relationship between increased nursing experience and lower pain prevalence in residents, and a relationship between the years of staff experience and the utilization of non-drug methods to treat it, being more frequently used by those with greater pain experience and training (IncS 95).

Experience tended to affect judgements: Alm & Norbergh (IncS 3) found that nurses with less experience had less confidence in making a judgement about pain themselves; however Baeza et al. (IncS 6) reported that care home staff with more experience tended to feel more stressed when not knowing the cause of pain for residents with dementia, and thereby less able to make their own judgement.

One systematic review (IncS 5) reported a correlation between less experienced staff and the verbally disruptive behaviour of residents, which may have been a manifestation of untreated pain.

Findings were also reported relating to staff turnover, type of working contract, and relationships with pain assessment and management, with evidence that pain assessment and management was performed better when there was lower staff turnover, and staff were hired permanently, and had longer tenure time (IncS 5, 16, 29, 39).

Care homes where staff had higher qualifications appeared to perform better in pain assessment and management, and give better quality of care (IncS 14, 15, 100). In addition, while some GPs lacked knowledge about pain assessment themselves, they valued the role of nurses and other caregivers (IncS 53), even although some staff perceived GPs as disinterested in relation to pain (IncS 78).

Care home staffs’ beliefs and perceptions exerted an influence on pain management, as examined in 40 of the included papers. For example, several papers reported that when staff felt more confident about the identification of pain, they were more certain in relation to its management (IncS 10, 44, 62). However, when staff considered pain to be part of ageing, they were less likely to identify pain in a resident (IncS 62). It was also reported that those residents who thought that pain was part of getting older were influenced by staff attitudes towards treatment, potentially resulting in reduced therapy options (IncS 104).

There was evidence that many staff did not agree with the statement that pain is part of aging and cannot be treated (IncS 8); however, with regard to residents with dementia, 50% of staff *did* consider pain as part of aging in this population (IncS 14).

When exploring nurses’ knowledge and attitudes to pain assessment in people with dementia, Burns & McIfatrick (IncS 13) reported that even amongst those with very recent training in pain assessment, it was still considered to be a “guessing game”; such beliefs had the potential to result in a higher prevalence of untreated pain in residents with dementia (IncS 94). However, when staff were familiar with residents, they found it easier to interpret non-verbal cues of pain (IncS 49, 61, 68, 104), and potentially differentiate it from other forms of distress (e.g. emotional distress).

Staff beliefs and concerns about the safety of using opioid analgesic were highlighted in several studies (IncS 9, 14, 53, 59, 76). These concerns related to either addiction beliefs, or fears about increased confusion and sedation; drug utilization was also reduced when either nurses or residents refused to use or take certain medications, even when their use may have been benefical.

There were some findings relating to staff perceptions about why residents might not report pain: for example in one paper it was suggested that staff were unsure if residents were displaying pill-seeking behavior because they wanted attention, rather than being truly in pain (IncS 30). In other papers, it was felt that residents might not be reporting pain because they were concerned about potential loss of independence (IncS 61, 76) or did not want to be seen as complainers or difficult residents (IncS 59, 61, 104).

There was some evidence from relatives that related to the approaches of staff to pain management, and also relatives’ feelings of being involved in the care of their family member. For example, Barry et al. (IncS 7) found that relatives who visited care homes more often considered that pain was being noted and treated to a greater extent by the staff. Also, relatives visiting their relatives frequently were more likely to interpret (and report to staff) behavior changes as signs of pain (IncS 37), and thereby felt more actively involved in their care (IncS 24, 59).

### Pain assessment

Of the included studies, 16 examined the use of pain assessment tools; in general these did not appear to be widely utilized (IncS 10, 31, 52), with some GPs in particular having little knowledge of specific tools to assess pain in care home residents (IncS 53). In one of these studies, detailing results from 810 participants across 7 European counries, it was reported that 58% of staff did not use any tool to assess pain in their daily practice (IncS 108). In other studies, although staff had knowledge of the tools, they reported problems interpreting them (IncS 97, 109), or felt them to be time consuming (IncS 42, 79, 84), However, when staff were trained in the utilization of tools, their use was more likely (IncS 97), and with increased usage, staff appeared to recognise their value to a greater extent (IncS 72).

The impact on residents of tool usage was highlighted in several studies. For example, the proportion of residents with pain appeared to increase, because pain questions were being asked (and recorded) more frequently, and also pain was being treated to a greater extent (IncS 74, 83). The use of tools was considered to increase awareness, and render staff more sensitive and responsive to residents’ pain-related behaviour (IncS 69).

The most commonly researched tool in the included studies was the Pain Assessment in Advanced Dementia scale (PAINAD). Where used, pain assessment tools that were found to be helpful included the Pain Assessment Checklist for seniors with Limited Ability to Communicate (PACSLAC), DOLOPLUS-2, Pain Assessment in Non-Communicative Elderly Persons (PAINE), and the Pain Assessment for the Dementing Elderly (PADE). In contrast, the Abbey Pain Scale, while commonly used in practice, was found to be inaccurate when assessing pain intensity compared with staff-estimated pain intensity, and it was also considered to be lacking in direction about precision of usage (IncS 94).

### Interventions

Of the 22 studies examining pain treatment interventions, most were shown to produce effective results. Where pain management practice improvements were made, analgesic and non-pharmaceutical treatment use increased and pain scores were shown to reduce (e.g. IncS 19, 36, 87). However, a systematic review (IncS 89) reported that pain scores deteriorated (increased) where there was a high turnover of staff, and lack of physician support.

Over half of the pain intervention studies (*n* = 13) were related to non-pharmaceutical treatments, and many indicated that alternative therapies had the potential to be effective (IncS 20, 28, 36, 62, 101). Non-pharmaceutical treatments, which included massage, aromatherapy, exercise, psychological support, and humour therapy, were viewed favourably by staff (IncS 1), but not necessarily by relatives (IncS 1, 79). Managerial support and staff training for non-pharmaceutical treatments were seen as necessary to facilitate usage (IncS 1, 42, 60). However, a large study by Lukas et al. (IncS 71; participant *n* = 1900) indicated that moderate to severe pain was more likely to be treated by pharmaceutical means, and less likely to be treated with non-pharmaceutical or alternative therapies.

Among the 21 included papers that considered pain management for care home residents with cognitive and/or communication impairment, there was general acknowledgement that the recognition of pain was challenging (IncS 2, 6, 14, 18). There was also recognition that these residents’ pain may be less well treated (IncS 35, 44, 75), especially where cognitive impairment was more severe (IncS 35). A lack of staff knowledge (IncS 9), inadequate assessment (IncS 27, 46, 75, 77), or availability and appropriateness of pain education programmes were influencing factors (IncS 14, 24). However, training programmes, as further detailed below, could help to increase awareness (IncS 40, 43, 84), as well as improving recognition of behavioural and non-specific manifestations of pain for people with cognitive impairment (IncS 99).

Pain training interventions were examined in 21 papers, and were shown to have the potential to be effective in improving pain management and treatment use/appropriateness (IncS 19, 32, 33, 39, 40, 43, 63, 79, 84, 87, 97). As well as improved pain management, resident quality of care and quality of life also had the potential to be enhanced (IncS 11), which could have an impact on their longer term wellbeing.

There was some suggestion that increasing staff knowledge regarding pain management could reduce anti-psychotic drug use (IncS 17). Training was also helpful in the use of non-pharmaceutical treatments (IncS 19, 79), helping to improve staff awareness and confidence (IncS 102). There were also indications that training for non-registered care staff might be beneficial (IncS 51, 96).

### Consultation with stakeholders

In addition to the scoping review findings, visits to 16 care homes across one region in SE Scotland took place. Staff were unaware of the findings from the literature review. However, these conversations confirmed many points from the literature, with all care home managers expressing the need to improve pain assessment and management. Three quarters of the care homes had registered nurses on-site. Most care home managers recognised the presence of pain in the majority of their residents. One care home, where all residents had a diagnosis of dementia, had all residents on regular paracetamol. There was, however, an overall lack of knowledge about identifying and systematically treating pain, and a reluctance to use pain assessment tools; where they were used, the tools of choice were the Abbey pain scale and Doloplus2. The main barrier to their completion appeared to be a lack of knowledge about them, with staff saying “we just have to ‘pick it up’ from other staff”.

Distressed behaviour in residents with advanced dementia appeared to be more attributed to the condition rather than the possibility of the presence of pain. While a wide variety of pain medication was used (e.g. non-opioids, anti-inflammatories, and opioids), there was still some expressed concern about over-dosage. Nonpharmacological interventions were used, but only to a limited extent, and not at all in some care homes. The majority of staff had received no pain training. Where training was in place, it appeared to be on-line rather than face-to-face, limiting discussions with peers about experiences. Nonetheless, there was a desire to improve, although current external support systems did not appear to be very responsive with offering support.

Findings from the field research carried out at the care homes is summarized in Table 3.

**Table 3:** Summary of findings from stakeholders

Findings from stakeholders	
• Findings suggested that care managers grappled with the complexities of managing pain, but were keen to know how to better manage pain for their residents • Those care homes using a tool were using the Abbey Pain Scale, albeit with some reluctance • Mangers and staff felt there was a lack of consistency, with no clear pathway for the systematic assessment and management of pain • Staff were reluctant to use pain assessment tools, and their use was therefore limited. They found them complex to use. • For residents with dementia, challenging behaviour appeared to be more attributed to the condition, rather than the possibility of the presence of pain • While a wide variety of pain medication was used (e.g. non-opioids, anti-inflammatories, and opioids), there was still some expressed concern about over-dosage • Non-pharmacological interventions were used, but only to a limited extent, and not at all in some care homes • The majority of staff had received no training in relation to the assessment and management of pain • A desire to improve was expressed by most managers and it appeared that both internal and external contexts needed to be strengthened to achieve this.	

## Discussion

This review has examined evidence relating to pain relief in care homes, and sought to understand contexts that might influence the assessment and management of pain for residents. As such, it fulfils the requirement of a scoping review to map the current state of evidence, rather than produce a detailed critical appraisal [11]. To our knowledge, such a broad scoping review has not been previously undertaken.

By including multiple research designs and methods, the review gives a comprehensive overview of findings from a diverse range of research studies. It updates, incorporates, and broadens the focus of the 13 included reviews, which examined narrower perspectives (e.g. management of specific types of pain, such as musculo-skeletal: IncS 34; or cancer-related pain: IncS 90; and reviews specific to dementia: IncS 14, 50, 76, 80).

Care home staff knowledge and training (or lack of it) occurred as a common thread across the different themes identified in the review. It was clear that training staff in the assessment and management of pain, and training in the use of appropriate pain assessment tools, in addition to experience and knowledge of residents, did reap benefits for residents in terms of greater comfort and quality of life, and helped staff to identify treatment options. However, a report by the International Longevity Centre (ILC) [14] argues that a lack of funding for training above and beyond essential or mandatory training exists. It is unclear what regulatory bodies for care homes in other countries (if in existence) advise; however, in the UK, recent reports published by the Care Quality Commission (CQC) show little or no focus on pain [15]. Where training does take place, it is often focussed on the need to fulfil other mandatory CQC requirements, rather than a commitment towards workforce development [14]. Prescribing for older people generally is recognised as complex, and staff education is essential to improve outcomes [16]. Due to budget limitations, such training may need to be funded by external sources, in the interest of resident well-being, staff satisfaction, and overall care home performance. Areas that would benefit from training programmes for care home staff are summarised in Table 4.

**Table 4:** Training programme recommendations

Suggested items for training programme inclusion	
• Generic pain education, including the exploration of attitudes, barriers and beliefs (open to all relevant staff, including GPs, care assistants etc) • The use of analgesia in older people – dosages, side effects, and monitoring, including the use of opioids • Recognition and treatment of pain for people with cognitive impairment • The use and interpretation of pain assessment tools, and subsequent treatment • Non-pharmacological pain treatments instead of, or in conjunction with, analgesic medication	

Lack of training may account for reduced staff knowledge and/or concerns about the use of opioids for older people. The differing views of experts may add to this confusion: according to Guerriero [17], guidelines should recommend opioid use as a first line treatment for moderate to severe persistent pain in older adults, whereas the American Geriatrics Society [18] advise more caution due to the potential side effects (disorientation, potential respiratory suppression, constipation etc). The World Health Organisation (WHO) pain ladder, which advocates stepping up to opiod use if pain is not controlled, has not been validated for non-cancer chronic pain [19], and may contribute to inappropriate prescribing if used in non-cancer pain [19]. However, current NICE guidance [20] does not rule out opioid use if other drugs prove ineffective, and also advocates the use of non-pharmaceutical treatments to augment or compliment medication. More detailed guidance is clearly needed for staff involved in the care of older people with chronic pain, and may assist staff decision-making.

With further regard to staff training, Wright [21] argues for care assistants to have greater access to training, considering it a key factor in attracting and retaining suitable employees. This is consistent with WHO guidelines relating to health staff retention [22], which state that financial incentives alone are insufficient to retain staff. Frogner & Spetz [23] also take up the theme that improved education and training for staff, alongside higher wages, and higher overall staffing levels, are necessary to combat and lower staff exit rates. The high turnover of staff in care homes has been called the ‘elephant in the room’ by Wilson [24], given that approximately 25% of staff leave in any one year [14]. This makes sustainability of any improvement in the assessment and management of pain less likely, unless there is regional support for training and retention.

Unfortunately, working in the care home sector may not viewed as a career choice for many qualified nurses, even though there are many more beds in nursing homes than in acute care hospitals, and the care needs of residents are increasingly complex [25, 26]. Whilst the difficulty of recruitment and high turnover of staff is therefore frequently acknowledged as problematic, the evidence from this review shows a correlation between high turnover of staff and less well controlled pain; this would appear to contravene a duty of care. It has also been suggested that older people cared for at home may have less pain and greater comfort than those being cared for in care homes [27].

On a more positive note, this review has also highlighted that more experienced staff, familiar with their residents, are more likely to include non-pharmaceutical measures in the management of pain (IncS 1, 20, 28, 36, 62, 101). That said, the use and effectiveness of non-pharmaceutical treatments can be aided by appropriate assessment prior to, and following, interventions.

It is evident that in order to overcome barriers relating to pain assessment, such as the use of tools to augment observational judgment, the mechanisms by which the integration of new practice occurs, need to be understood to a greater extent [28]. If staff can see the value and rewards associated with using a pain assessment and management framework (e.g. increased resident comfort), then shifts in attitudes and norms can occur more readily [28]. Pain in frail older people needs to become part of the regulation of care homes, although it has been argued that this may not happen until care homes adopt an electronic minimum data set or equivalent tool [29]; this has gained more interest recently, stimulated by conversations as a result of the Covid-19 pandemic [30].

Further points of relevance highlighted by the review include the need for clear communication and support from external professionals, including greater access to GPs, and the value of including the views of relatives regarding the detection of pain.

It could be argued that highlighting the skill required to deliver quality care to residents, and the importance of good pain assessment/management, may help to attract nurses and care staff into a career with frail older people in care homes. The value placed on such care by the public and politicians has certainly been highlighted during the COVID19 pandemic [31], and may provide a timely platform from which to launch recruitment drives.

### Review strengths and limitations

It is acknowledged that the review may not have captured all relevant material. Searches, screening, and study selection are all open to error or bias. However, the rigorous methods utilised, including blind screening and cross-checking, have served to minimise these limitations. The broad nature of the review, including seeking care home staff opinions, has enabled an expansive view of the available knowledge, rather than a narrower or confined perspective. With such a large volume of evidence being included in the reveiw, we acknowledge that another research team may have chosen to emphasise other areas within the included research. However, seeking the views of local care home staff did serve to validate the findings, and their interpretation, in this review.

## Conclusions

This review has highlighted that training and explicit guidelines for the appropriate assessment and treatment of pain remain a current requirement for care home staff. Knowledge of the issues that face care home staff and their residents can help other professionals, external to the care home environment, to target their input in the most appropriate way. Internal and external contexts need further examination in order to co-create a framework that integrates the assessment of pain and its management, to the benefit of patients.

In essence, there is a need for pain assessment and management training for all staff in care homes, including training relating to residents with dementia. Raising the importance of good pain assessment and management may alter staff perceptions relating to the presence of pain in older people, particularly those with cognitive impairment.

Increasing staff knowledge regarding pain management may reduce anti-psychotic drug use, and increase the use of non-pharmaceutical treatments.

The use of tools may also help to increase awareness, and render staff more sensitive and responsive to residents’ pain-related behaviour. However, pain assessment and its management may not improve in care homes if there remains a high turnover of staff. In addition, clear communication between care home staff and both relatives and external professionals is essential to promote identification of pain and its management.

## Supplementary Information

**Additional file 1.**

**Additional file 2.** [32–140]

## Data Availability

Further data and a fuller report on this review are available on request from the authors.
