# Correction to: Pain assessment and management in care homes: understanding the context through a scoping review

**DOI:** 10.1186/s12877-021-02413-5

**Published:** 2021-09-22

**Authors:** Jan Pringle, Ana Sofia Alvarado Vázquez Mellado, Erna Haraldsdottir, Fiona Kelly, Jo Hockley

**Affiliations:** 1 Scottish Collaboration for Public Health Research and Policy, University of Edinburgh, Edinburgh, UK; 2 St Columbas Hospice and Queen Margaret University, Edinburgh, UK; 3 School of Health Sciences, Queen Margaret University, Edinburgh, East Lothian UK; 4 Usher Institute, University of Edinburgh, Edinburgh, UK

**Correction to:** ***BMC Geriatr*** **21, 431 (2021)**

**https://doi.org/10.1186/s12877-021-02333-4**

Following publication of the original article [1], the authors identified an error in the reference list. The references of the main article and the supplementary files were combined into 1 list.

For clarity, the reference lists have been split. The original article has been updated.
