# Supplementary material for: Pain assessment and management in care homes: understanding the context through a scoping review
Source: BMC Geriatr. 2021 Jul 18;21:431. doi: 10.1186/s12877-021-02333-4 (PMC8286436; doi:10.1186/s12877-021-02333-4)
Supplement: Supplementary file 1 — Additional file 1. [file 12877_2021_2333_MOESM1_ESM.docx]

**Additional file 1: Medline (Ovid) search strategy**

1 exp Pain/

2 Myalgia/

3 Neuralgia/

4 Acute Pain/

5 Chronic Pain/

6 Hyperalgesia/

7 Neuritis/

8 Paresthesia/

9 (pain or myalgia or neuralgia or hyperalgesia or paresthesia or neuritis or soreness or ache* or discomfort or dys?sthesia or allodynia).ti,ab

10 Pain Management/

11 Pain Measurement/

12 Therapeutics/

13 Symptom Assessment/

14 Self Report/

15 (management or measurement or therapeutic* or assessment or self report or treatment or identification or recognition or detection or evaluation or appraisal or rating).ti,ab

16 (tool* or test*).ti,ab

17 Geriatric Nursing/

18 Nursing Homes/

19 Homes for the Aged/

20 (geriatric nursing or nursing home* or elderly institution* or geriatric nursing home* or care home* or residential care or aged care or aged care facilit*).ti,ab
