# Supplementary material for: Pain assessment and management in care homes: understanding the context through a scoping review
Source: BMC Geriatr. 2021 Jul 18;21:431. doi: 10.1186/s12877-021-02333-4 (PMC8286436; doi:10.1186/s12877-021-02333-4)
Supplement: Supplementary file 2 — Additional file 2. [32–140] [file 12877_2021_2333_MOESM2_ESM.docx]

| **Additional file 2: Included studies**  (abbreviations detailed at end of table) | | | | | | | |
| --- | --- | --- | --- | --- | --- | --- | --- |
|  | **Authors Date Country** | **Population** | **Study method/aim** | **Pain assessment tool(s)/ Intervention** | **Outcomes/ Variables** | **Findings** | **Authors conclusions** |
| 1 | Abrahamson (2015)  USA | 24 NHs employees, 8 facilities. | Qualitative case study;  to describe staff’s experiences in a quality improvement project aimed at reducing residents’ pain | Pain in Minimum Data Set; Quality improvement project | Experience of staff with the quality improvement project | Interdisciplinary communication, supportive leadership, training, and nursing assistant participation facilitates intervention. Increased documentation, resistance to change, and difficulty measuring outcomes were perceived as challenges. | Efforts to reduce pain scores could benefit from interventions designed to capitalize on the interdisciplinary expertise present in NHs. |
| 2 | Ahn (2015)  USA | 71 227 residents with dementia >=65 years old  73.6% female  Mean age 85 SD 7yrs | Secondary analysis of data; Relationship between pain and aggression in residents with self-reported pain vs staff-assessed pain-behavioural indicators | Self-assessment;  Numeric rating scale or Verbal descriptor scale;  MDS Pain Assessment Interview (verbal);  MDS Pain Behaviour Scale (non-verbal); No intervention | Pain;  Verbal and physical aggression | Pain self-report positively associated with verbally aggressive symptoms. Residents who can verbalize pain are more likely to receive treatment, reducing the odds of being physically aggressive. Lack of medication was associated with residents who could not self-report pain, for men and for those with severe cognitive impairment. | Staff should assess for pain in both verbal and nonverbal residents to improve pain management and reduce the risk for aggressive behavioural symptoms. |
| 3 | Alm (2013)  Sweden | 56 nurses, 92.8% female, mean age 46.8 SD 9.18; nursing working experience 18.28 SD 10.6 yrs | Cross-sectional;  To determine if patient’s nonverbal cues influence the nurses’ opinion of pain | Numeric Rating Scale; Measurement of pain in two scenarios that differ only in nonverbal cues (smiling or grimacing face) | Nurses’ pain report compared to self-report; medication given compared to what should have been given | Facial expressions can make pain identification harder. Many nurses did not assess the smiling patient as being in pain (even though they self-reported pain). Only 25% nurses shared the patient’s value. Nurses with less experience documented the patient’s self-reported value more often than more experienced nurses. | Resident is the expert of his/her pain and should be regarded as such. Nurses should be aware of the different ways of coping with pain. Good practice is talking to residents and asking about their pain, and using a NRS. (Mis) interpretation of nonverbal cues can lead to misunderstanding. |
| 4 | Alonso  (2013)  Spain | 10 residents (80% women). Intervention (n=5) (mean age 87 SD 2.44); control (n=5) (mean age 83.8 SD 3.82). | Pre and post-intervention; To design, apply and assess the efficacy of a psychological intervention program for musculoskeletal chronic pain | Brief Pain Inventory; Program based on acceptance and commitment therapy and training in selection, optimization and compensation strategies (SOC). | Functional performance; frequency of use of strategies, pain acceptance, pain and age beliefs, emotional well-being | Intervention group better acceptance of pain, and increased feelings of success for living in accordance with their own values. This group had a decrease in: catastrophizing pain as threating life condition; scores in disability and depression, and the belief that medication is the only treatment for pain. | Psychological treatment within acceptance and commitment therapy, and optimization/compensation strategies, may be an appropriate option for this population. |
| 5 | Anderson (2016)  Australia | 46 intervention papers and 35 predictor papers | Systematic review; To examine associations between staff variables, quality of care and QOL for residents | Pain as a QOL outcome; No intervention | Staff variables correlation with restraint and psychotropic medication and/or QOL | Restraint use and psychotropic use was associated with pain. Strong relationship between restraint use and staff fearing that residents will fall or be in pain. These fears linked to restriction of activity leading to reduced functional ability and more injuries because of falls. Staff distress and less care given to residents were linked to greater reliance on psychotropic drugs. | Staff need greater knowledge around dangers of restraint, and restraint use regulations. Where staff treat and interact empathically and humanely in care, there is a relationship with better affect for residents, delayed functional dependence, and better food intake. |
| 6 | Baeza  (2018)  Spain | 107 experts from 22 nursing homes. 79% female; mean age 36 SD 8.2 | Delphi study;  To know the opinion/beliefs of experts regarding pain in aging people with cognitive impairment | No pain assessment tools; no intervention | Opinions and beliefs regarding pain and methods to assess it | Disagreement with: pain just happens in the elderly, it cannot be treated, and it’s a way to feel alive. Agreement that people with cognitive/verbal impairment may receive inadequate treatment, and lack of assessment tools for them; also, experts use non-verbal cues and changes in behaviour to assess pain. Disagreement that pain may be equally evaluated in these people. | Staff are concerned about identifying and treating pain, especially in those with communication impairment. This concern is more marked in those who attend them more directly. |
| 7 | Barry  (2015)  UK | 42 residents (85.7% dementia); 16 nurses/care assistants; time since qualification 9.9yrs; experience in care homes 5.3 yrs. 35 relatives | Qualitative study; To determine pain frequency; variables associated, analgesic use; relatives views on pain management | No pain assessment tools; no intervention | Pain reporting; medication usage; variables associated with pain; relatives’ perception of pain and treatment | 88.1% of residents were prescribed with at least one analgesic, but it wasn’t regularly scheduled. Antipsychotic medication use was found to be associated with the presence of pain in residents with dementia. Relatives who visit more often their residents disagree more with the idea that pain was unnoticed or untreated by the care home. | The processes of pain assessment and management must be standardised within NHs to ensure residents in pain receive timely, good quality person-centred care. |
| 8 | Barry  (2013)  UK | 182 pharmacists, 59.3% female; mean age 36.8 years; 57.7% qualified > 10yrs, practicing for average of 13.7 yrs | Cross-sectional; To explore pharmacists’ experiences /attitudes towards dementia; knowledge about pain and its management | No pain assessment tools; no intervention | Experiences, attitudes, knowledge about pain in dementia | 28.6% respondents said pain is a consequence of ageing (more likely in those with training in pain).  68.7% agreed dementia can affect psychological processing of pain.  46.7% agreed that perception of pain in dementia is different.  64.8% were unsure if people with dementia were more likely to experience pain than those without it. 95.6% had not received training in dementia; 93.4% no training in pain. | Education and training should be developed for pain assessment and management in residents with dementia, to improve and enhance pharmacists’ knowledge and attitudes. |
| 9 | Barry  (2012)  UK | 96 NH managers (nurses qualified for an average of 24.9 years), 89.6% female | Cross-sectional; To explore managers’ knowledge, attitudes and beliefs about pain in residents with dementia; | No pain assessment tool; no intervention | Knowledge, attitudes, beliefs regarding pain assessment and management | 60.4% of NHs use pain guidelines.  Results related to: pain experience,  Pain assessment, management and treatment: harder to assess in people with dementia (91.7%), management should be the same for any resident (57.3%). 78% disagree with “harmful nature of painkillers”. Barriers included: obtaining an accurate report of pain from the resident’, ‘lack of staff knowledge/education about pain management this residents and ‘lack of a standardised approach to treating their pain. | Improved training regarding pain in people with dementia and implementation of standardised guidelines for pain management are needed. |
| 10 | Ben Natan (2013)  Israel | 104 nurses, mean age 38.8 yrs SD 8.9; 82.7% female; duration of nursing career 15.9 yrs SD 10.2; experience 13.16yrs SD 8.6 | Cross-sectional; To explore knowledge about pain assessment | No pain assessment tools; no intervention | Pain assessment practices and attitudes in older people, association of variables | Participants with intention to assess pain, but few actually do it. No correlation between pain training on the job and intention to perform an assessment neither between attitudes about pain assessment and intention and actual performance of it. Attitudes towards older people, and the perceived control over pain assessment were predictors of nurses’ pain assessment performance. | There is a need to increase pain assessment training as part of pre-service nursing education. It should focus on improving attitudes towards older adults, removing negative myths, and increasing appreciation of the importance of pain assessment. |
| 11 | Bird  (2016) Australia | 46 intervention papers; 35 predictor papers | Systematic review; To describe staff interventions and its impact on quality of care or resident quality of life | No pain assessment tools; No intervention | Quality of care; residents’ quality of life | Common interventions were: training in pain recognition and its assessment using standardized tools; psychosocial/drug management protocol for residents with severe dementia; training to recognise discomfort and associated behaviour; and identifying individual learning needs of the staff and ways to meet their/residents’ needs. | Aiming at a specific target may be more useful than more generic interventions. Didactic education needs mentoring/support. Interventions need several months of follow-up (both staff and residents variables). Studies should: have larger samples, sensitive measurements, measures applicable to the clinical target. Interventions with staff are intermittently effective. |
| 12 | Boerlage (2013) Netherlands | 201 residents; 60.7% female | Cross-sectional;  To determine the feasibility of pain assessment as performance indicator in quality of care | Numeric Rating Scale (NRS), a Verbal Rating Scale (VRS), or the Rotterdam Elderly Pain Observation Scale (REPOS);  No intervention | Pain prevalence and analgesic prescription | 45% residents with substantial pain received pain medication of step 1 of the WHO analgesic ladder; 12% received weak opioids (step3), and 5% received a neuroactive agent. 38% with pain did not get any medication. 4% received opioids with NRS ≤3. Those with NRS ≥4 had more analgesics than those with lower NRS. | Caregivers and physicians should have training on pain on a regular basis. Pain assessment is a feasible performance indicator. Pain assessment combined with a pain treatment algorithm makes a good combination for the improvement of pain treatment. |
| 13 | Burns (2015a)  UK | 32 nurses in 17 NH; 84.4% female; mean age 41yrs; average of 8.6 yrs’ experience working in NHs | Cross-sectional; To determine nurses’ knowledge/attitudes towards pain assessment in dementia. | No pain assessment tools; no intervention | Factors influencing knowledge/attitudes towards pain assessment; training; barriers to pain assessment | 68% used pain treatment guidelines. Abbey pain scale was the most identified. Barriers to pain management: limited staffing, lack of interdisciplinary support and workload pressures. Nurses with training had significantly higher scores in relation to pain assessment and management. Needs in education: knowledge of analgesic choice and better understanding pain assessment tools. | Need to develop pain education programmes specifically designed for nurses caring for older people with dementia in order to improve knowledge in the effective assessment and management of pain. |
| 14 | Burns (2015b)  UK | 11 studies published | Systematic review; to explore nurses’ knowledge/attitudes to pain assessment in dementia | No pain assessment tools; no intervention | Attitudes and knowledge | Results included: challenges in diagnosis pain in dementia, inadequacies of pain assessment tools, communication and interdisciplinary teamwork, time constraints and workload pressure, and training and education. Nurses identified a lack of availability and appropriateness of pain education programmes. | The accessibility of appropriate training, workforce stability and a standardised approach to pain assessment are key to the successful management of pain in residents with dementia. Research is required to examine practical concerns relating to interpretation and clinical use of pain assessment tools in dementia. |
| 15 | Castle (2015)  USA | 3913 administrators; mean age 51.2 SD 10.1; 22% female; 33.2% with master/higher degree, and 55.3% with bachelor degree; mean experience as administrator 12.2 SD 7.4 years | Cross-sectional; To examine association between administrator’s education and quality of care; association between state educational/ training requirements and quality of care | No pain assessment tool; no intervention | Association between education of administrators and nursing home care indicators | Administrators with more advanced educational background were associated with better pain management. Higher educational requirements and higher training requirements are associated with better quality of care. | Promoting further educational attainment of administrators may influence the quality of care. State educational requirements and training requirements for administrators’ licensure can also influence quality of care. |
| 16 | Castle (2011)  USA | 3941 nursing homes | Longitudinal analysis; To examine relationship between staffing levels, turnover, agency use, and professional staff mix with quality of care | No pain assessment tools; no intervention | Quality of care indicators (pain management, included) | Negative correlation with pain management: higher levels of staffing; low levels of agency staffing in nurses aids, and registered nurses; decreasing levels of turnover of nurses’ aides and registered nurses; and, increased levels of professional staff mix. Lower level of turnover of nurses, and decreasing levels of agency staffing of licensed practical nurses, didn’t have a significant correlation with pain management. | Improving staffing characteristics can improve quality of care. Longitudinal studies, quarterly staffing data, and larger sample sizes facilitated reaching this conclusion. |
| 17 | Cervo  (2012)  USA | 215 participants; mean age 84.9; 52.1% female | Non-randomized pre-post intervention; To implement and evaluate the CPAT tool, and changes in scores | CNA Pain Assessment Tool (CPAT); Incorporation of CPAT in long-term care pain management clinical practice guideline on nursing home residents with dementia. | Number of falls, episodes of distressed behaviour and rates of psychotic usage; CPAT score changes | Not significant reduction of Verbal Aggression behaviour after intervention. Antipsychotic use was significantly reduce. CPAT scores significantly decreased in the post-nondrug and pharmacologic treatment group. The CAN PAT is useful to assess effects of pain treatment in this population. | Pain assessment tools need to be included in pain management guidelines, in order to provide benefits. In this study this resulted in reduction of falls and a decrease of antipsychotic medication in residents with dementia. Recommendation of assessing pain on a daily or more structured basis, instead of the 2-3 times per week used here. |
| 18 | Chang (2009) Australia | 5 Focus groups (diverse health staff); 13 interview participants from 10 NHs. | Participatory Action Research; To expose the challenges of care for people with dementia | No pain assessment tools; no intervention | Needs and deficits in service delivery | Several participants reported that pain management is difficult, with assessment as a major concern. They agreed that with dementia, non-verbal signs are very important to observe. A nurse acknowledged that agitation observed in a resident could be due to discomfort from pain. | Improved knowledge and skills, and a model of care that accounts for the diverse symptoms and presentations of dementia are needed. |
| 19 | Chen  (2016) Taiwan | Experimental: (n=98), mean age 82.7 SD 8.2, 50% female. Control (n=97), mean age 83.6 SD 7.2, 60.8% female | Cluster-RCT (3 months follow up); To compare the effect of a Pain Recognition and Treatment protocol coupled with basic education | Verbal Descriptor Scale, PAINAD, Cohen-Mansfield Agitation Inventory; Pain recognition and treatment protocol coupled with basic pain education (experimental) versus basic pain education alone (control) | Pain management performance; pain-related expression of residents with dementia post intervention and at 3 months of follow up | Intervention group used significantly more non-drug strategies and referrals at post intervention. It did not have significantly more weekly pharmacologic strategies at all, and had significantly lower weekly average pain scores in the follow up. Control group had significantly more weekly pharmacological strategies and referrals at follow-up, and significantly higher weekly pain. | A systematic problem-solving approach and the cooperation with other healthcare professionals can help nurses become more responsive to the pain of residents with dementia, and empower them to make changes in their practices. |
| 20 | Cino  (2014)  USA | 118 participants, 75% female, mean age 83 | RCT; To examine a nursing intervention for chronic pain | Geriatric-Multidimensional Pain and Illness Inventory factors; Iowa Pain Thermometer; Effect of aromatherapy hand massage compared to massage without aromatherapy; or just nurse presence | Pain; pain intensity; pain suffering; life interference; emotional distress | Hand massage with or without aromatherapy provided a reduction in chronic pain intensity, compared to the presence of nurse only. | Utilisation of hand massage as a potential treatment for chronic pain in older adults is suggested. |
| 21 | Cohen-Mansfield (2015)  USA | 89 residents with dementia, 73% female | Cross-sectional; To describe unmet needs of residents with dementia. | Pain Assessment in Elderly Person (PAINE); no intervention | Unmet needs | Main unmet needs: boredom/sensory deprivation, loneliness/need for social interaction, and need for meaningful activity. With better cognitive function there are less unmet needs of pain. Discomfort is related with greater pain and higher levels of verbally agitated behaviours. PAINE tool is useful to estimate pain in people with dementia. | There is a need to develop programs that aim to address unmet needs, especially social and activity needs for residents, and to improve detection and treatment of physical needs that result in pain and discomfort. This could enhance person-centred care and quality of care. |
| 22 | Cohen-Mansfield (2014)  USA | 89 residents with dementia; mean age 85.9 SD 8.62; 73% female | Cross-sectional; To examine the influence of personal characteristics and barriers on the efficacy of nondrug interventions in reducing agitation | PAINE;  Interventions provided based on an individual algorithm | Observed agitation (with the Agitation Behaviour Mapping Instrument) | Residents with dementia that can speak have greater improvement in agitation when having nondrug treatment. Those with higher cognitive function had lower levels of agitation, and fewer difficulties to perform activities, speech, communication, and responsiveness. Staff barriers (e.g. refusal) decrease the effect of intervention on agitation. | Maintaining verbal communication with residents can optimize their care and quality of life. Health care staff must be receptive to suggestions regarding pain and discomfort. |
| 23 | Corazzini (2010)  USA | 678 staff in 16 long-term care settings (9 nursing homes); 96.6% female; mean age 40 SD 13.1 | Cross-sectional; To examine relationship of contextual/ organizational factors with training outcomes of a dementia care program | No pain assessment tool; Foundations of Dementia Care Training Program | Knowledge, communication, pain awareness; work stress related to work | Supervisors had more baseline knowledge than staff of racial and/or ethnic minority status. Older staff showed less increase in knowledge of pain awareness after training compared to younger participants. Laissez-faire administrative climate was related to lower baseline pain awareness. | Facilities’ administrators should consider the administrative climate, communication patterns, and the need for training before starting a training program. |
| 24 | Corbett (2016)  UK | Care home staff, family carers | Mixed methods; to explore current landscape of pain management in people with dementia | No pain assessment tools; no intervention | Pain management | Abbey Pain Scale is commonly used. Paracetamol is the first option, and staff dubious of using opioids. Staff rely on medical notes and residents’ charts, but there is no clarity on how they are used. Pain assessment dementia is difficult. Difference in education and barriers in communication between different members of staff. Staff need to be more confident and have more responsibility to assess pain. | Evidence-based pain management programs are needed. They must be informed by stakeholders and based within a conceptual framework for this particular setting. |
| 25 | Cranley (2012)  Canada | 50 health care aids, 2 senior decision-makers, 4 nurses/care coordinators, and 14 managers/educators. | Qualitative study; to identify areas for a project improvement | No pain assessment tools; no intervention | Areas of improvement | Areas of care for improvement: pain/discomfort management, behaviour management, depression, skin integrity, and assistance with eating. | Involving staff in the areas that they perceive as needing improvement can aid their engagement in the process. |
| 26 | Damsgard (2018) Norway | 8 nursing, physiotherapy pharmacy and medicine students (87.5% female) | Qualitative study; To pilot project to improve student comprehension of pain in NHs residents | No pain assessment tools; Educational project to improving pain understanding and management | Inter-professional learning | Important to know the patient to understand and manage pain. Pain can be associated to lack of social activity. Pre-existing knowledge and experience of staff are crucial for choosing treatment. It is very important team knowledge sharing and common reflections on observation; and that the whole team knows the patient personally. | Health care curricula should include knowledge of pain management in residents of NHs, and the practice of inter-professional cooperation. |
| 27 | De Souto Barreto (2013)  France | 6275 residents from 175 NHs; mean age 86 SD 8.2; 73.7% female | Cross-sectional; determine whether dementia and the absence of pain assessment reduce analgesic use | Visual analogue scale or other instrument; no intervention | Analgesic use | People with dementia (OR 0.75, 95% CI 0.66-0.85) and those who don’t have any pain assessment records (OR 0.64, 95% CI 0.53-0.79) are more likely to not receive analgesics. | It is recommended to include systematic pain evaluations in NHs routinely, to improve pain management. |
| 28 | Decker (2012) USA | 20 residents; mean age 83.6 years; 90 % female | Quasi-experimental; feasibility of healing touch intervention to treat pain | Verbal Descriptor Scale and Pain Assessment Tool in Cognitively Impaired Elders; Healing Touch intervention vs Presence Care Intervention | Pain, activities of the daily life, quality of life | Mean pain/discomfort scores decreased from pre to post intervention for both groups, but it wasn’t significant. | Healing touch is a feasible intervention for residents with pain. |
| 29 | Decker (2010)  USA | 787 NH administrators and 703 Directors of nursing; mean job tenure (years): administrator (6.56 SD 0.24), directors (4.67 SD 0.18) | Cross-sectional; To examine association of top management with the prevalence of pressure ulcers, pain, and physical restraint use. | No pain assessment tools; no intervention | % of low-risk residents with pressure ulcers, % of residents experiencing moderate to severe pain, and % of residents with physical restraint | Increasing the administration tenure was significantly associated with a decrease in pain prevalence. A year increase was associated with a -0.74% change. An increase in directors of nursing tenure was significantly associated with a -1.12% reduction. Tenure of 10 years or more had a significant change of -14.1% relative to that at 1 to 2 years tenure. | Stability of top management in nursing homes can have an impact on better residents’ outcomes. |
| 30 | Dobbs (2014)  USA | 28 certified nurse assistants; mean age 38.1 SD 10.5; 96.4% female; experience 12.5 SD 9.1 years. | Qualitative study; To explore pain management | No pain assessment tools; no intervention | Communication, empathy towards pain, effect of race and ethnicity in pain experience | Pill-seeking behaviour is thought to be personality-dependent, and can be seen as a way to look for attention. Pain can be told to relatives more easily; it can be communicated until it’s unbearable. Residents communicate more easily with staff of their same gender and ethnicity. Empathy increases with experience. NAs can use guessing, questioning, or touching to identify pain. | NHs need a person-centred approach to pain that is culturally competent, given the cultural influences of both staff and residents. |
| 31 | Dockerty (2016)  UK | 408 managers of 14 care homes | Cross-sectional; To explore musculo-skeletal pain management | No pain assessment tools; no intervention | Pain assessment and treatments | 93% use more than one method to assess pain; only 29% use a formal score chart. 71% monitor pain in a daily basis. 93% used multiple methods to treat pain (painkillers and physiotherapy as basic treatment). | Authors recommend more research to explore why electronic surveys have very low response rates. |
| 32 | Douglas (2016)  Australia | Baseline: 216 staff /74 residents; experience in aged care of <2 years (16.2% vs 16.5%); 3-4 years (23.8% vs 16.1%), > 5 years (60% vs 67.4%) | Pre-test/post-test study; to evaluate a pain identification tool | New tool (different strategies combined); Pain identification tool and workshops | Staff-knowledge and attitudes, confidence and skills for pain assessment; frequency of pain assessment and use interventions; residents’ perception of quality of pain management | Small improvement in knowledge and attitude at the end. Increase understanding that pain is not part of aging. Increase in documenting routine pain. Increase in number of therapies for residents. Improvement in residents’ perception of the quality of pain management. Tool was helpful and improved quality of pain management. Increased pain awareness, its identification, and staff communication. | Using pain identification resources, along with implementation strategies, is partially effective to improve outcomes in both residents and staff. |
| 33 | Drager (2017) Germany | Intervention (n=100), control (n= 95); mean age 83; 72.3% female. 12 NHs | RCT; To evaluate effects of an intervention on pain intensity and pain interference with function | Modified German version of the Brief Pain Inventory; Separate training measures for nursing staff and physicians using different delivery methods | Pain intensity; pain and pain interference with function, relations with others, sleep and enjoyment of life. | Intervention group: lower pain intensity, and pain interference with function, specifically walking. Significant reduction in pain interference with mood, walking, ability to cope and enjoyment of life. One quarter perceived an increase in pain, this was lower in the intervention group (22.2% vs 27%). | Authors recommend intense and long-term interventions in order to train nursing staff. |
| 34 | Dragerset (2014)  Norway | 11 included studies | Systematic review; to understand assessment and management of cancer-related pain in residents with/without dementia | No pain assessment tools; no intervention | Pain assessment and management | Residents >85 years, and those with dementia tend to receive less analgesia and opiates than younger ones. Pain behaviour negatively associated with Alzheimer’s. Relatives or residents can see pain as a metaphor for illness and death. | There is an urgent need for better guidance and evidence on pain management for residents with severe dementia. |
| 35 | Dube  (2018)  USA | 367, 462 residents with cancer and cognitive impairment; 52.3% female | Cross-sectional; to evaluate variations in pain documentation and management in residents with cancer and cognitive impairment | Minimum Data Set (MDS); no intervention | Pain and pain management | Prevalence of pain and its treatment (non-drug or pharmacological) is lower in residents with dementia (more marked in more advanced dementia). Self-assessment may be the best option for those who are cognitively intact and less effective with severe cognitive impairment. | There is a need to improve techniques, treatment, and documentation of pain in residents with cognitive impairment. |
| 36 | Ellis  (2019) Australia | 95 residents; mean age 83 SD 7.6; 62% female | Retrospective study; To evaluate a non-drug pain management program (massage, exercise, stretching, nerve stimulation) | Pain numerical rating scale, direct questioning; Abbey Pain scale; Non-drug treatment 10 minutes/day for 4 days a week during 8 weeks | Pain scores; pain medication | Statistically significant reduction in pre-session pain, no change in post-session pain over the 8 weeks. Lower pain after sessions than pre-sessions (statistically and clinically significant). Residents without dementia had slightly better improvement. Massage and nerve stimulation decreased pain more than other treatments. | Non-drug pain treatments may be effective to reduce pain and reliance on medication, especially when two or more are used together. |
| 37 | Eritz  (2011) Canada | 81 informal caregiver/resident dyads. 9 facilities. | Cross-sectional; To clarify the pain decoding process by determining the types of nonverbal cues that informal caregivers use to evaluate pain in loved ones with dementia | Coloured Analogue Scale; PACSLAC; Video recording of residents during potentially painful routine daily activities, and this was shown to their informal caregivers | Interpersonal Reactivity Index | Caregivers didn’t interpret non-verbal pain cues as pain behaviours, but spending >10 hours with resident made them to be more likely to consider nonverbal behaviour into account. Caregiver’s empathy, depression, age, sex didn’t predict pain ratings. Caregiver pain ratings were only related if resident was moved from resting to a moving condition. | It is important to develop interventions that focus on training caregivers to be more aware and attentive to specific pain cues, especially in residents with dementia. |
| 38 | Ersek  (2016)  USA | 27 NHs; 485 residents (259 intervention (mean age 83.9 SD 8.3, 70% female) | RCT; to enhance pain practices using pain assessment and management algorithms and intense diffusion strategies. | Pain thermometer scale; Iowa Pain Thermometer; Intensive training for the use of pain assessment and management practices using algorithms | Nursing assistant report and self-reported resident pain intensity; adherence to recommended pain practices. | NA’s pain reporting improved slightly at the end. Pain self-reported improved, but it wasn’t clinically significant. Adherence outcomes improved in both groups. Assessment score improved (not significantly) in intervention group. The intervention was no better than basic pain management education. | Research needs to focus on finding methods to implement interventions in a more effective way. |
| 39 | Ersek  (2014)  USA | 4 nursing homes; 24 staff (with NH experience of 10 years in average); 92 resident’s charts | Mixed-method study; To examine facilitators and obstacles to adopt evidence-based pain management protocols | Pain Management Chart Audit Tool; Intervention to promote the adoption of evidence-based pain management protocols | Perspectives, facilitators and barriers to changes in pain assessment and management practices. Changes in pain assessment/management | Enablers: involvement of directors, policies and procedures, audit mechanisms and individual accountability, implementation in phases, education and availability or resources, multidisciplinary and consistency of staff. Barriers: physicians’ mistrust of nurses’, staff’s fears to drugs, lack of time and high ratio of residents-to-nurse. Benefits: more frequent pain assessment, increase confidence to report pain. | In order to change clinical practice in NHs is necessary to increase attention to both structural and process aspects of care. |
| 40 | Fine  (2014)  USA | 8 facilities; 5 physicians, 33 registered nurses, and 13 administrative employees. | Intervention (non-RCT); To enhance pain management through an education activity | Numeric Pain Intensity Scale, Visual Analog Scale, MDS Target Assessment, Pain Faces Scale, Verbal Rating Scale; A performance improvement continuing medical education activity | Pain; cause of pain; care plans; effectiveness of pain management | Performance improved (larger when having a care plan). Better recording of pain medications, their efficacy, and safety; special attention given to non-verbal, confused, or agitated residents. Health care staff other than doctors were often responsible for pain-related care. Pain assessment on admission increased. Numeric Pain Intensity Scale, Visual Analog scale, and MDS Target Assessment use increased from baseline; Pain Faces Scale, and Verbal Rating Scale use decreased. | Interventions should focus on the interactions between physicians and other health care professionals, such as nurses. Also, programs should aim to obtain institution and/or system involvement. |
| 41 | Flaig  (2016) Germany | 40 Nursing homes, 560 residents, mean age 81.2, 60.9% female | Cross-sectional; to assess frequency of physician contacts with residents and the influence in the appropriateness of pain management. | No pain assessment tools; no intervention | Number and type of physician contacts, appropriateness of pain medication; influence of physician contacts on the appropriateness of pain medication | The frequency of physician contacts correlated weakly with the appropriateness of pain medication (p = .039). Every physician contact is related with more appropriate medication (p = .056.) | Research should evaluate demand, orientation, and quality of physician contacts in nursing homes. |
| 42 | Gagnon (2013)  Canada | 148 staff; mean age 45.35 SD 10.62, 92.6% female; mean years in current job 18.4 SD 11.6, mean years working in LTC 14.24 SD 9.56 | Mixed methods; To develop and evaluate a training video on pain assessment | No pain assessment tools; Training video on pain assessment | Beliefs about pain, knowledge gain, appreciation of the video | Training was considered useful and relevant, but hardly put into practice. Barriers: insufficient time and staff, co-worker negativity and resistance to change. Enablers: manager’s involvement, constant support, and foreseeing benefits of implementation. Challenges: not trusting in pain self-report and care aides’ pain report, incomplete and inconsistent charting, family demands, and physician interference with pain management. | Top –down implementation approaches with ongoing management involvement throughout the implementation process may be needed to achieve sustained changes in pain assessment. |
| 43 | Ghandehari (2013)  Canada | 131 nurses and special care aides, mean age 44.1 SD 12.5; 17.6 SD 11.8 years of experience in health care, and 12 SD 9.5 years working in LTC | RCT; to investigate effectiveness of an education program in pain assessment/management | No pain assessment tools; An education program in pain assessment/management for LTC staff | Pain knowledge and beliefs, acquisition of pain-related knowledge | After intervention, the following increased: pain knowledge, confidence to overcome barriers, vigilance, use of non-drug strategies, and use of medication under regular schedule. Positive aspects: interactive instruction, experts’ participation, small group talks, content, proposed tool (PACSLAC), diversity of treatments. Negative aspects: too many information. Barriers to implement it: lack of time, other staff and their communication. | Pain education in nursing homes has the potential to address knowledge gaps in health care professionals working in long term care facilities. |
| 44 | Gilmore-Bykovskyi (2013)  USA | 13 nurses from 4 skilled nursing facilities | Qualitative study; to examine how nurses make decisions to pharmacologically treat pain in residents with dementia | No pain assessment tools; no intervention | Decision-making | Certainty of pain determines pain management. When uncertain: delays in treatment, and it is more common in long-term stay residents, dementia, pill-seeking behaviour, no obvious reason for pain. When certain: more in those dying, less delays in treatment. With dementia, nurses try different treatments on “trial an error”, based on an individualized approach. Withdrawn or disengaged residents are less likely to get analgesics. | Interventions aiming to improve decision making among nurses should include education and also address the need for additional system supports and facility-level integration of appropriate pain management practices. |
| 45 | Griffioen (2017) Netherlands | 324 elderly-care physicians and 111 trainees; mean age 45; 67.7% female | Cross-sectional; To evaluate degree of knowledge of opioids among elderly care physicians, and attitudes toward its utilization | No pain assessment tools; no intervention | Knowledge of opioids, barriers for its clinical use, and attitude-related factors for its use | 87.7% participants agreed that the risk for dependence on the use of opioids is not a problem when they are prescribed in the correct way. Barriers to use: patients’ not willing to take them, and pain unknown origin. 44.4% participants considered fear for the development of delirium as side effect. | There is a need for information regarding pros and cons of opioid use. Research examining the reasons why residents don’t want to use opioids should be conducted. |
| 46 | Gropelli (2013)  USA | 16 nurses; mean age 41 years; 93.8% female; nurses were licensed an average of 15 years, and average time employed was 11 years. | Qualitative study; to investigate nurses experiences with pain management | No pain assessment tools; no intervention | Nurses’ perceptions of pain management | Nurses’ pain beliefs: caused by anxiety, residents are not able to cope with pain. Participants recognized a lack of education on pain management, especially when residents can’t verbally identify it. Nurses try first non-drug measures, and then medication. Nurses acknowledged: need of individualized plan of care, lack of staff communication, written communication with physicians is effective, but verbal one is lacking; relatives can think nurses just want residents to be quiet with drugs. | Pain management programs need to focus on nurses’ perceptions and biases. Nurses need to understand their perceptions in order to assess and address their effects. Nurses need more pain education. |
| 47 | Gudmanns-dottir  (2009) Iceland | 12 residents, ages, mean age 86 years; 41.6% female | Qualitative study; to examine the experience of residents in chronic pain in NH | No pain assessment tools; no intervention | Barriers successful pain management | Barriers to pain management: thinking it has no cure, passive attitude, hiding or resigning to it. Some feel proud when not complaining. Residents can have too much faith in the number of pills they take. Residents felt nurses distant, and associated them with bringing the pills, but not with pain management. | Multidisciplinary collaboration with education is needed towards quality pain management of elderly people in NHs, where existential pain and suffering is included. |
| 48 | Guion  (2018) France | 3277 residents; mean age in strong intervention residents (86.5 SD 9), in light intervention (87.4 SD 9). | Non-RCT (18 months); To determine efficacy of an educational and professional support intervention to improve pain management | yes/no question; Intervention based on education and professional support (strong (auditing, feedback and collaborative work between physician and staff) light (auditing and feedback only) | Pain complaint, pain-related covariates; pain management (gold standard or not, using a pain scale and/or no pain medication) | Strong intervention group had a lower number of people complaining about pain at 18 months; residents with a gold standard in pain management significantly increased. | Nonspecific, collaborative, educational, and organizational interventions are useful to improve pain management. |
| 49 | Holloway (2009) Australia | 6 nursing assistants from 3 facilities; employment duration 6 months to 23 years | Qualitative study; to explore experiences of NAs in aged care facilities to ascertain their role in the facility | No pain assessment tool; no intervention | Experiences in aged care facilities, | NAs assess, document, and start pain treatment (PRN). They are close to residents and this allows them to identify pain signs. NAs decide course of action either communicating to nurses or ignoring the problem. Sometimes they start treatment and check effectiveness. NAs described a role of advocacy for residents. | NAs need preparation to initiate and implement pain management treatments. This group of workers need regulation as they are performing clinical tasks in NHs. |
| 50 | Husebo (2016) Norway | 12 included studies | Systematic review; to determine pain assessment tools for people with dementia and their responsiveness to pain intensity, analgesics, | No pain assessment tool; no intervention | Pain assessment tools for people with dementia; indicators of pain and pain behaviour; pain medication | Pain Assessment in Non-communicative Elderly persons (PAINE) and Pain Assessment for the Dementing Elderly (PADE) pain tools were the most responsive to assess pain intensity in residents with dementia. The Mobilization-Observation-Behaviour-Intensity-Dementia (MOBID)-2 Pain Scale is responsive to a decrease in pain after analgesic treatment. CMAI was responsive to pain medication. | There is a need for research with well powered pain medication trials in persons with moderate-severe dementia using pain assessment tools that have been tested for responsiveness. |
| 51 | Hyer  (2010)  USA | 6 residents (age 73.2 SD 12.5), and 5 nursing assistants (age 44.6 SD 12.46, mean experience at the current NH: 12.2 SD 7.39 years). All female | Pilot study; To evaluate the feasibility of a nursing-home-based pain coping skills training intervention | Brief Pain Inventory (BPI) Short Form; pain coping skills training intervention (relaxation, imagery, pleasant activities, and activity-rest cycling) | Self-efficacy, pain, discomfort, and activities of daily living (ADL) function | Residents increased self-efficacy scores after the intervention. Nursing assistants’ self-efficacy scores decreased. Pain and its interference with daily activities (especially with walking) decreased after intervention. | Pain coping skills may help to reduce pain in severely disabled nursing home residents. |
| 52 | Jablonski (2009)  USA | Medical records of 291 residents of 14 facilities; mean age 86.6 SD 7.76; 81% female | Retrospective study; To determine the extent to which staff adhere to evidence-based guidelines to assess and manage pain | Pain Management Chart Audit Tool; Iowa Pain Thermometer; no intervention | Pain (intensity and location), pattern and character | Low adherence to evidence-based pain assessment guidelines. Pain assessment was found in 85% of records. Lack of documentation regarding pain location, intensity, pattern, character, and impact on quality of life; and drug’s side effects. Efficacy of as needed medications was assessed more frequently than those routinely given. Nondrug therapies were just documented in 11% of charts. | Staff and administrators must critically examine both systems and individual staff reasons for failure to comply with best pain management practices. |
| 53 | Jennings (2018)  Ireland | 108/157 questionnaire answered by GPs; years of experience: 12.7% between 0-5 yrs.; 30.6% 6-15 yrs.; 26.1% 16-25 yrs.; 30.6% >= 26 yrs. | Cross-sectional; To explore GP’s knowledge and attitudes towards pain assessment and management in people with dementia | No pain assessment tool; no intervention | Knowledge and attitudes regarding pain assessment and management in dementia | 60% do regular rounds to the NH. They agreed on the difficult to assess pain in dementia; agreed on importance of observing behavioural changes. 10% knew pain tools for these residents, and 14% knew pain guidelines in the NH. GPs agreed on stepwise approach to treat pain. Not sure about opioids. They are not sure of using pain assessment tools, as it would increase workload. They recognize value of nurses and other caregivers. | It is necessary to identify aspects of pain care in residents with dementia, to design effective interventions to appropriately address their pain. |
| 54 | Jordan (2015)  UK | 43 residents from 5 sites; mean age 78.7 SD 11; 58.1% female | Cluster RCT; to assess clinical impact, potential cost impact, and harms of nurse-led medicines’ monitoring | No pain assessment tool; Structured nurse-led medicines’ monitoring (West Wales ADR Profile for Mental Health Medicines) versus usual care | Problems addressed and changes in medicines prescribed, individual problems, disease severity, potential costs and harms of intervention | Whit intervention pain was more constantly found. When following the Profile, nurses were more likely to document and treat pain. The intervention was feasible, low cost, low risk, and convenient. | It is necessary to examine effects of structured medicines’ monitoring on clinical outcomes, nurses’ workloads, and bridging the gap between patients and prescribers. |
| 55 | Jordan (2011)  UK | 79 residents with dementia; mean age 82 SD 8.14; 72% female | Prospective study; to evaluate the utility of a distress tool and a pain tool in a population with dementia | PAINAD, and DisDAT for distress; Individual interventions according to their assessment | Pain score; distress signs and behaviours | There was a significant improvement in pain with both tools (PAINAD and DisDAT) after a month of intervention. Many of the signs and behaviours of distress were the same in the group with real pain, and in the false positive group, even though the underlying cause was different. | Pain tools can also pick up distress, which is not caused by pain, and can lead to false recognition of pain. Distress tool picks up a broader range of signs, which may be useful in both practice and research. |
| 56 | Jordan (2010)  UK | 79 residents with dementia; mean age 82 SD 8.14; 72% female | Prospective study; To investigate the utility of PAINAD tool in those with advanced dementia in NHs | PAINAD and DisDAT for distress; Individual interventions according to their assessment | Pain score; distress | PAINAID is a sensitive tool to assess pain in residents with dementia, but has a high false positive rate, which can be caused by psychosocial distress rather than pain. False positives could have happened because participant didn’t understand the situation and felt anxious, frightened, frustrated or angry. | PAINAD is a useful tool to assess whether pain management strategies have been successful. |
| 57 | Kaasalainen (2015)  Canada | 2 care homes; and 2 nurses with more than 10 years of experience | Exploratory, multiple-case design study; To explore the role of a clinical nurse specialist and nurse practitioner as change champions during the implementation of a pain protocol | No pain assessment tool; evidence based pain management protocol | Activities and processes related to implementation of the protocol; barriers to implement it | Having a champion to implement a pain management protocol improves the efficacy of the implementation. Barriers to the protocol: it was too rigid, extra work, not taken as a priority, timing, and difficulties to train all staff. Facilitators: it’s effective to have a champion; it was helpful to teach other staff; and, support provided by administrative staff. | It is important to implement changes through knowledge transfer and exchange in the NHs, and there must be a recognition of the influential role of certified nurses and nurse practitioners. |
| 58 | Kaasalainen (2012)  Canada | 99 residents in intervention (mean age 82.5 SD 9.43; 52.5% female), and 101 in control group (mean age 83.6 SD 7.5; 76.2% female) | Controlled before-after design and qualitative study; To evaluate the effectiveness of dissemination strategies to improve clinical practice, and the implementation of the pain protocol in | PACSLAC, the Pain Assessment in the Communicatively Impaired (PACI) Elderly, and the Present Pain Intensity Scale; implementation of a pain protocol using a multifaceted approach | Pain, use of a pain assessment tool, documentation of pain management, use of pain medications | Improvement in the use of a standardized pain assessment tool, and initial pain assessment. Important to remind staff of pain as a priority. Having advanced practice nurses and a pain team contributed to success of intervention. Pain increased significantly in the control group. | Interventions should include all team members, residents and family members. The intervention and its implementation need to be refined to optimize current practices and resources available to staff. It also must be housed within an effective knowledge exchange approach to promote sustainability. |
| 59 | Kaasalainen (2010)  Canada | 53 staff, 71% female; mean age 49.3 SD 13.54; average experience in current position 8 SD 7.6 years, and in LTC for 11 SD 9.93 years | Case study approach; To explore barriers to pain management, and to develop an inter-professional approach to improve it | No pain assessment tool; evidence-based pain protocol intervention | Barriers to pain management and to implement a program in LTC | Barriers: lack of resident’s self-report, lack of time to assess pain, relatives’ concerns regarding medication, unregulated care staff don’t feel supported in their role, lack of communication between staff; pharmacists consider hard to treat pain as they don’t know residents, neither if the medicine worked; pain assessment is not a priority in orientation sessions, lack of training and education, extra workload. | Efforts to address barriers to optimal pain management need to focus on various levels of the health care system in order for pain management programs to be fully realized. |
| 60 | Kalinowski (2015)  Germany | 239 residents; mean age 82.9 SD 8.1; 70% female; 12 NHs | Cluster RCT (6 months); to assess non-pharmacological therapies, and to enhance their application | 2 Dichotomous question and the Brief Pain Inventory (BPI); Pain management interventions (e.g. online course for physicians and 1-day seminar for nurses) | Pain severity, appropriateness of pain medication and non-pharmacological therapy provided | After the intervention there was an increase (but not significant) in the utilization of non-drug treatments applied by nurses in the intervention group. | Residents must receive education on how to manage their pain to support them in taking a proactive role in managing their pain. |
| 61 | Knopp-Sihota  (2019) Canada | 31 included studies | Systematic review; to report barriers and facilitators to pain assessment in nursing home | No pain assessment tool; no intervention | Barriers and facilitators for pain assessment. (residents, health care provider, health care system factors) | Resident factors: cognitive status, resident’s behaviours and attitudes towards pain; residents care about sex and ethnicity of staff. Staff factors: lack of knowledge/skills, familiarity with resident, experience, confidence and sense of control, attitudes and behaviours (better assessment when pain is not taken as part of aging). System factors: poor interdisciplinary work, low staffing levels, high turnover, lack of time and funds. | Identifying barriers and facilitators to pain assessment have the potential to improve the identification of pain in residents, and may improve its management. |
| 62 | Knopp-Sihota (2016) Canada | 14 trials included; 2293 participants at baseline (mean age 83 years, 70% female) and 2029 participants at the end | Systematic review and meta-analysis; to assess efficacy of pain-reduction interventions | No pain assessment tool; no intervention | Analgesic and non-analgesic treatments, pain scales, efficacy of interventions | System modification interventions had effects at the beginning but not at the end of trial. Education-based interventions didn’t have benefit. Electro-acupuncture and transcutaneous electrical nerve stimulation significantly reduced knee pain. Light physical exercise, watching humorous movies, whey protein supplementation, and volunteer visiting significantly reduced pain perception. | Some non-analgesic treatment benefits, but analgesics should be considered first-line therapy, as they are the most effective when treating pain. |
| 63 | Konner (2015) Germany | 6 NHs in control group and 6 NHs in intervention group. 239 residents; mean age 82.9 SD 8.1; 70% female | Cluster-RCT; to evaluate the effect of interventions for GPs/staff to improve pain and appropriateness of pain medication | Numeric Rating Scale; educational intervention | Average pain severity and appropriateness of pain medication (PMAS) | Intervention group reported a non-statistically significant increase of the PMAS score at the end of the intervention. The score of PMAS for each individual in the intervention group showed a statistically significant increase at the end of intervention. | Utilisation of real time prompts and alerts (intervention tools) should be used to assess effectiveness of interventions. Interventions should consider participations of staff and GPs together to strengthen their collaboration. |
| 64 | Lapane (2012)  USA | 9849 residents from 174 nursing homes; age range (<50 - +85 years) | Quasi-experimental study; To estimate if guidelines’ revisions improve pain recognition and management | MDS 2.0; revisions to the surveyors’ interpretive guidelines for F-Tag 309 | Pain assessment and management | Pain and scheduled analgesic prescription was more likely to be documented after the revisions were implemented. | It is recommended to use directed language to stimulate improvement in pain documentation and management. |
| 65 | Lautenbach-er  (2017) Germany | 79 nursing homes, 284 residents (mean age 78.5 SD 9.6) ; average 14 years health care working | Cross-sectional study; To investigate features of facial expressions caregivers rely when assessing pain in people with dementia | Facial descriptor items from established observational pain assessment tools for people with dementia ; No intervention | Pained expression, pain intensity | Frowning, opened mouth, and looking tense were found to be significant predictive items for pained expression and pain intensity. Nurses observe anatomically based descriptors (frowning and narrowed eyes), and indicators of emotional arousal (looking tense/frightened). These signs predicted the overall pain ratings. | Future development of pain assessment tools based on observation should consider the pre-existing assumptions that nurses use to infer pain, their competence to infer it, as well as their potential bias. |
| 66 | Leone (2009)  USA | 40 residents with dementia from 2 skilled care and dementia units; mean age 91.4; 84.2% female | Cross-sectional; To analyse baseline pain level, implement a pain scale, and identify challenges for its adoption | Visual Analog Scale (1-5 and with faces), PAINAD; Introduction of pain scales and training sessions for its administration scales | Pain level, number of narcotics and other analgesics used, updating of standing orders | Patients with moderate to severe cognitive deficit had higher levels of mild pain. For them it was difficult to answer the verbal pain scale. Staff adopted successfully the tools, found them helpful and adequate to assess pain. Nurses’ participation in tailoring the scales, and giving feedback during implementation was seen as a facilitator of success. | It is recommended to have regular cognitive and behavioural assessment to help evaluate pain. It is important to train all staff, have supporting policies, and proper tools and strategies to have successful programs. |
| 67 | Liu  (2017)  Hong Kong | 128 residents with advanced dementia from 17 NHs; intervention n=64 (mean age 89.3 SD 7.11 ; 88.4% female); control n=64 (mean age 87.87 SD 7.6; 70.8% female) | Cluster-RCT; to investigate whether the implementation of the Observational Pain Management protocol improve pain management in residents with dementia | PAINAD Chinese version; Implementation of the Observational Pain Management Protocol (12 weeks) | Use of pain medications and non-pharmacological pain treatments (types and frequency), pain score | Intervention group had a significant increase in the frequency and type of non-pharmacological interventions; and significant reduction in observational pain score. No significant change in pharmacological treatments. | It is recommended to daily use, and document, observational pain assessments and treatments. This is a good way to increase staff awareness of the need for pain treatment. |
| 68 | Liu  (2013)  Hong Kong | 49 nursing assistants from 12 NHs. 95.92% female. | Qualitative study; to explore nursing assistants’ roles in pain management for residents with cognitive impairment | No pain assessment tool; no intervention | Experiences in pain management | NAs work close to residents, and this allows them to identify pain, and to know what makes residents feel comfortable. NAs can feel powerless and submissive as they feel nurses don’t support them. They don’t feel they are considered in pain management decision. NAs don’t perceive assessment of medication effectiveness important. They feel confident with non-pharmacological interventions. | Nursing assistants have supporting roles in pain management. It is suggested to use a pain management protocol and create coherent work teams to improve pain management. |
| 69 | Liu  (2012)  Hong Kong | 30 cognitive impaired residents; mean age 82.1 SD 7.8; 46.7% female.  48 staff; mean age 38.34 SD 8.07; 95.83% female; employment 5.6 SD 5.24 years | Exploratory study; To report development and implementation a Pain Protocol, and its impact on pain management; | PAINAD Chinese version; Implementation of the Observational Pain Management Protocol (12 weeks) | Pain intervention; opinions | Non-pharmacological interventions increased at the end of the intervention; pain decreased slightly. NAs felt tool made them more sensitive and responsive to residents’ pain, and to be more systematic when assessing. NAs felt protocol helped to convince nurses quicker about identification of pain. | It is suggested to train staff on Observational Pain Assessment measures to ensure they perform a structured and formal pain assessment. |
| 70 | Liu  (2011)  Hong Kong | 77 staff; mean age 45.75 SD 8.03; 88.31% female; employment 8.96 SD 5.24 years | Cross-sectional; To explore staff acceptance of using Pain Behaviour Observational Methods (PBOMs) | PBOMs; no intervention | Clinical relevance of pain indicators, self-appraisal of using PBOMS, | Facial expressions were the most observed, followed by eye movements, vocalisation and verbalisations. Staff felt familiar, confident and comfortable with observing behaviours. Staff felt PBOMs lacked standards for interpretation, and that they can increase workload. | Observing non-verbal pain cues is something informally and regularly done by nurses. It is suggested to educate and create guidelines on the use of PBOMs to aid regular and systematic implementation. |
| 71 | Lukas  (2013) Germany | 1900 residents; mean age 83.9 SD 9, 75.6% female | Cross-sectional study; To identify pharmacological and non-pharmacological pain management approaches across Europe | InterRAI instrument for Long Term Care Facilities (interRAI LTCF); no intervention | Pain; pain medication factors associated with different pain management approaches | Being female, cancer, and moderate/severe pain was positively associated with pharmacological pain management. High staff turnover, and low/moderate physician’s availability were negatively associated. Non-drug treatments were positively associated with fractures and need of assistance in daily living activities. Dementia, larger facilities, high turnover, low physicians’ availability, and severe pain were negatively associated. | European nursing homes need improvement regarding pain assessment and treatment. |
| 72 | Mamhidir (2017)  Sweden | Intervention (n=130) mean age 85.5 SD 6.7, 71.5% female. Control (n=83) mean age 85.1 SD 7.2; 63.9% female. 56 staff (RNs and NAs) | Cluster-RCT and mixed methods; to investigate effects and experiences of a pain management intervention | NRS-scale for self-assessment and Doloplus-2; Intervention group (theoretical and practical training sessions, application of observational scales mainly) | Wellbeing and proxy-measured pain, ADL-dependency and pain documentation | 29.5% of residents in intervention had lower pain at the end. Nurses considered Doloplus-2 adequate to capture pain in dementia. Expression and sleep patterns were useful. Familiarity with residents helped pain identification. Staff considered scale as time consuming, but they recognized its value. Pain assessments facilitate more effective teamwork and drug treatments. | Increased awareness, collaboration, and shared understanding among staff members of pain assessment results can improve pain management. |
| 73 | Monroe (2015a) USA | 43 residents, median age was 84 years, 67% female | Pre-test/ post-test; To determine if specific order sets related to pain assessment improves pain management | Direct question from the nurse, 7-item interview derived from the Geriatric Pain Measure; Implementation of specific order sets for pain assessment | Observed nurse pain assessment queries, resident reports of pain, functional ability | Nurses asked about pain more frequently, and continued doing this after one month after intervention. Proportion of residents with pain increased, because it was assessed more frequently. Residents just communicated pain when they were asked directly about it. | Using specific order sets seems to improve the recognition of facility-level pain prevalence. |
| 74 | Monroe (2015b) USA | 29 RNs and Licenced practical nurses | Qualitative study; to assess NH personnel`s clues and practices to identify and alleviate pain in residents with dementia | No pain assessment tool; no intervention | Cues and practices for pain assessment and management | Assessing pain in dementia takes longer and is challenging. Nurses focused more on the comfort of the resident. Factors that help to identify pain: nurses’ clinical expertise, family, familiarity with resident, and interdisciplinary communication. Struggle to find balance between family’s wishes and resident’s needs (pain). | Nurses promote measures to increase comfort and improve quality of life, focusing on family and the resident, and this helps pain assessment in dementia. Future work should focus on comfort care qualities, and how nurses decide on the use of pain medications vs psychotropic agents. |
| 75 | Nakashima (2019)  USA | 50 673 residents. Mean age 83.2 SD 8.9; 75.5% female; 68.4% with dementia | Cross-sectional (data from 2012); To compare pain interventions received by residents with/out dementia | Minimum Data Set 3.0; no intervention | Pain interventions (scheduled pain medication, PRN pain medication, non-medication intervention) | Residents with dementia had less pain assessment, and less reported pain presence than those without dementia. Residents with dementia were significantly less likely to receive both non-pharmacological and pharmacological treatment when needed. | It is recommended to design a pain assessment tool that can be sensitive to differentiating severity of pain, so appropriate interventions can be applied. |
| 76 | Newton (2014)  UK | Literature about adults with dementia | Review | No pain assessment tool; no intervention | Pain management and pain assessment | Pain in dementia is poorly identified. Some patients don’t report pain as they feel there is nothing to do, or they afraid of medication. Residents > 80 years are more reluctant to admit pain. Staff believe older people can’t tolerate opioid medications; that not expressing pain means an absence of it, and that pain perception decreases with age. | It is suggested to have a multi-dimensional perspective that centres on the individual when assessing and managing pain. |
| 77 | Osterbrink (2014)  Germany | 151 nurses and NAs; 12 facilities; 81% female. | Ex-post-facto design; To evaluate pain assessment and to identify need for improvement | No pain assessment tool; no intervention | Written pain policies and procedures, pharmacological and non-drug approaches, | Nursing homes have pain policies, and they asses it regularly (not for severe cognitive impairment). Requirement of pain assessment documentation is largely met, but written policies are missing. 50% of nurses evaluate pain at admission. 98% ask about location, 75% about intensity, and 50% about frequency. 67.5% reassess pain regularly. 50% of NHs had policies on pain reassessment schedule. | Pain management guidelines should include a detailed and explicit reassessment schedule for the different needs of residents. |
| 78 | Peisah (2014) Australia | 15 facilities. 20 staff members | Cross-sectional; To explore attitudes and experiences of staff regarding pain management in residents with severe dementia | No pain assessment tool; no intervention | Attitudes and experiences | Frequency of pain assessment more regulatory-driven, than patient-driven. Staff commonly looked for nonverbal cues of pain. 35% reported behaviour changes as a trigger for pain assessment. Paracetamol main treatment when pain was observed, and occasionally with behaviour changes. All but one facility provided pain education once or twice a year. Non-drug treatments are used sporadically. Failure in communication between staff. | Early, proactive, consideration and management of pain behaviours is recommended for people with dementia. Also, empower nurses as interpreters of residents’ needs, and create collaborative partnerships with common goals between residents, family and staff. |
| 79 | Petyaeva (2018)  UK | 3 facilities with 36 to 63 residents. | Feasibility study with nested resident case studies; To stablish the feasibility and initial effectiveness of training and support a pain intervention | Abbey Pain Scale, MOBID-2, and Pain Interference Scale for Dementia; Training and support intervention in pain management in people with dementia (PAIN-De) | Feasibility, pain management behaviours; pain, goal attainment for the resident related to pain management | Opposite opinions regarding intervention in 2 focus groups. FG1: increase in confidence and awareness of pain, staff using more non-verbal cues, utilisation of person-centred approaches, and acknowledged value of following intervention. FG2: lack of time and motivation to use non-drug approaches, no understanding of person-centeredness approach, wanted to pass responsibility to more senior members. | In order to have an impact on staff behaviours and successfully implement a pain management protocol it is important to involve all the staff, including managers. It would be beneficial to also include family members to maximise impact. |
| 80 | Pieper (2018)  Netherlands | 21 Clusters in 12 nursing homes; 288 residents. Intervention (n=148) (mean age 84.3 SD 7.4; 72.3% female). | Cluster-RCT (6 months); To assess a stepwise multidisciplinary intervention and its effect in pain | PACSLAC- Dutch version, MDS-RAI; Stepwise multicomponent intervention vs control group (training on general nursing skills, dementia management and pain without stepwise component) | Observed and estimated pain, pain medication | The intervention had an overall effect on observed pain but not on estimated pain. Opioid use increased, paracetamol use did not. The intervention improves the assessment and management of pain in addition to challenging behaviour. | Addressing behavioural cues for pain in dementia may be essential for better pain management. A systematic, stepwise approach is superior to knowledge, training, or implementation without a systematic, stepwise approach. |
| 81 | Pieper (2013)  Netherlands | 16 included studies | Systematic review; to provide evidence regarding effectiveness of pain interventions in behaviour, and behavioural interventions in pain, in residents with dementia | No pain assessment tool; no intervention | Interventions targeting pain or behavioural in dementia | Pain interventions targeting behaviour: pain medication reduces disruptive behaviour. Interventions targeting both pain/behaviour: reflexology, multimodal cognitive behavioural therapy, rocking chair therapy, and towel bath method were identified as causing a decrease in pain and behavioural changes. | Better pain assessment and management, preferably individually tailored, may be an effective strategy in managing challenging behaviour. An approach targeting both pain and behaviour is recommended. |
| 82 | Rababa (2018)  Jordan | 76 residents, mean age 75.52 SD 9.45, 44% female. 22% with severe dementia | Descriptive design; To examine nurse’s level of pain certainty, assessment scope, and pain outcomes | Observational visual analogue tools (Discomfort-DAT); no intervention | Pain, nurse’s certainty, comorbid burden, ability of residents with dementia to verbally self-report symptoms | Nurse’s certainty of pain and the different methods used to measure pain were moderately negatively correlated. As dementia became more severe, the scope of assessment increased. Pre assessment level of nurses’ certainty, assessment scope and post assessment level of nurses’ certainty are predictors of pain outcomes. | It is important to understand the relationship between nurses’ certainty, assessment scope, and patient outcomes for people with dementia. |
| 83 | Reid  (2015)  USA | 89 health care staff | Pre-test/ post-test; To improve the ability of caregivers to recognize, assess, and manage pain | PACSLAC, Abbey Pain Scale; Quality improvement initiative (educational workshop for staff, and survey) | Effectiveness (baseline, 3 and 8 months after workshop). Adequate pain assessment and management | After intervention there was an increase in: confidence to identify nature and causes of pain; pain documentation, nonverbal cues utilisation and documentation, and, non-drug approaches. Prescription of opioids decreased. Baseline to 3 months: significant increase in the utilisation of PACSLAC, but decreased at 8 months post-intervention. | A quality improvement initiative is an effective way to improve pain care practices. Education plus multidisciplinary activities are needed to achieve sustained improvement. It is advised to perform repeated assessments of performance to identify educational needs. |
| 84 | Rodriguez (2018)  USA | 32 health care staff in training | Quality improvement project; Training NAs to use PAINAD for dementia, and to improve staff communication | PAINAD; training in the use of PAINAD (3 months) | Tool (ease of use, confidence level, ability to identify behaviours; appropriateness, feasibility and relevance of training); pain | Training was effective, and tool was described as easy to use. Videos were useful to understand training. Some thought that additional paperwork might discourage to continue using the tool. Most frequent recognized behaviour was negative vocalization. | Using PAINAD in the electronic medical record can promote direct communication between staff. It is suggested to track interventions, their completion, and reduce paperwork burden. |
| 85 | Rostad (2018) Norway | 16 NHs; 112 residents with severe dementia; mean age 83.6 years; 69.9% female | Cluster-RCT; to assess if regular pain assessment is associated with changes in pain and analgesic use | Doloplus-2; experimental group had regular pain assessments twice a week for 12 weeks | Pain score and analgesic use | There wasn’t a change on pain score or analgesic use compared to the control group. | It is necessary to explore how, when, and for whom, pain assessment tools can be used to support pain treatment in residents with severe dementia. |
| 86 | Russell (2010)  USA | 26 facilities in both intervention and control groups | Secondary analysis data; to ascertain if intervention, improves pain management | Different pain assessment tools; to build good system care practices and leadership practices to improve staff performance and resident outcomes | Quality measure / quality indicators pain scores | Intervention group didn’t show a greater improvement in pain management scores compared to the control group (just education). NHs that focused in pain management during their intervention improved in pain management documentation, and follow up for effectiveness. | Multifaceted approaches and interventions that focus on education, implementation of revised care practices, and quality improvement monitoring strategies, are needed. |
| 87 | Savvas (2014a) Australia | 5 facilities; 282 residents mean age 85 years; 77% female | Pre- post-test design; To review pain management practices and to implement an evidence-based pain management program | Abbey pain scale, PAINAD, NOPPAIN; staff training and education and revised in-house pain management procedures | Pain scores; medication to treat pain | Analgesic use improved significantly after the program. It decreased in residents not receiving analgesics, and it increased in resident’s receiving around-the-clock plus as-needed analgesics. Significant improvement in pain scores. | Best evidence-based practice can be achieved with training, education, and changes to institutional pain management practice. It is recommended to invest resources in workforce. |
| 88 | Savvas (2014b) Australia | 5 facilities; 365 residents assessed at pre-audit and 330 assessed post-audit | Cross-sectional study; To address implementation of standards and evaluate outcomes | Resident’s Verbal Brief Pain Inventory, and Abbey Scale, or PAINAD; staff education and training, regular pain assessment procedure, pai n champions, and coordination of resources | NHs Self-efficacy in pain management, understanding of standards | Facilities improved compliance with standards. Staff reported significant better understanding of the standards, guidelines, increased their confidence in pain assessment and management, especially in residents with dementia who are nonverbal. | Appropriate training and allocation of resources to the workforce make it possible to achieve best evidence-based practice in NHs. |
| 89 | Shropshire (2018)  USA | 11 studies in meta-analysis, 2 in qualitative synthesis; 4055 participants, mean age 82.3 SD 7.4 years; 71.2% female | Integrative review; to evaluate state of non-pharmacological interventions for elders | No pain assessment tool; no intervention | Pain scores, quality of | Results about different Interventions for physical comfort (e.g. exercise, aromatherapy), psycho-spiritual comfort, sociocultural comfort (e.g. humour therapy significantly improved pain and psychological variables), and for environmental comfort (e.g. high turnover of staff, low availability of physicians and inadequate pain relief strategies were negatively associated with pain). | It is recommended to incorporate non-pharmacological interventions for pain management in long-term care. |
| 90 | Smith  (2016)  UK | 24 included studies; 263 775 residents in 12 countries; | Systematic review; to describe prevalence, impact and management of musculoskeletal pain in older people | No pain assessment tool; no intervention | Prevalence and frequency of musculoskeletal disorders. Staff’s attitudes and experiences towards these disorders, care pathways | Multicomponent assessment and management packages empower staff to manage pain, but don’t change clinical outcomes. Pain management programme can improve residents’ happiness. Cognitive behaviour therapy (with education/coping skills) reduced pain and pain-related disability in residents with and without dementia. Barriers: limited knowledge, not following guidelines, poor support inter-staff support. | It is important to understand diagnostic causes for pain, especially in older people. |
| 91 | Stacpoole (2015)  UK | 5 dementia care homes; 37 residents, (30 finished) | Action research study; To evaluate the effects of a programme on the behavioural symptoms of residents with advanced dementia | Doloplus-2; nursing programme that integrates compassionate care with individualised meaningful activities | Behavioural symptoms, and pain management | Severity of behavioural symptoms and occupational disruptiveness significantly decreased after the initiation of the programme in four CHs but increased in one. There was a significant positive correlation between pain and symptoms severity. | Nursing homes with strong leadership and good nursing and medical care facilitated the effectiveness of the program. |
| 92 | Swafford (2009)  USA | 10 included studies | Literature synthesis; to present process-level pain management projects and identify and describe the role and skills of staff in successful interventions | No pain assessment tool; no intervention | Pain management interventions; effects | Factors that improve pain management: systematic implementation of models, clinical decision-making algorithms, interdisciplinary approach, continuous evaluation and documentation of assessment practices and outcomes, collaboration between facilities, assessing pain in a regular way, and using scales according to residents’ needs; staff needs education, skills regarding pain but also organizational practices. | To improve pain practices it is important to have easily accessible, concise and uncomplicated Quality Improvement (QI) models and easy to use evidence-based resources. Nurses should assume leadership roles when participating in QI activities. |
| 93 | Takai  (2014)  Japan | 88 residents (mean age 84.54 SD 7.2, 75% female, ˃50% dementia); 23 staff (65.2% female, working experience 11.4 SD 7 years) | Feasibility study; To examine the feasibility and clinical utility of the Abbey Pain Scale, and staff opinions | Abbey Pain Scale; application of the scale | Interrater reliability, nurses’ and care workers’ opinions on use of the scale | Nurses and care workers use observational points of the scale to evaluate pain. Negative opinions: criteria not clear; difficulties in observing residents; need to practice, insufficient guidelines. Positive opinions: size, and ease of use. Clinical utility: gap between estimated pain intensity and scale score. | In order to facilitate the utilization of tools, education and clinical guidelines for pain management are required for all staff. |
| 94 | Takai  (2013)  Japan | 439 nursing ward managers; 94.1% female, mean working experience 25.2 SD 9.9 years | Descriptive study; To examine nursing ward managers’ perceptions of pain prevalence and pain management strategies | No pain assessment tool; no intervention | Perceptions about pain prevalence, and strategies for its management | 27.8% assess pain at admission, 12.2% assess pain continuously. Enablers: education, utilization of manuals, and availability of medical treatment or diagnosis by physicians. Managers’ age and nursing experience were related with lower pain prevalence. Believing that pain is natural and finding time as a constraint is related to higher pain prevalence. | Appropriate pain management guidelines, dissemination, and education, are needed to improve practice. |
| 95 | Takai  (2009)  Japan | 31 nurses, 92 care workers, and 18 residents with chronic pain in 8 facilities | Cross-sectional; To assess the frequency of chronic pain care approaches, and to assess the factors that influence this | SF-36 v2 (includes bodily pain); no intervention | Frequency of use of chronic pain care approaches, factors affecting its utilization | Frequent approaches: gently handling and support while providing care, listening attentively, and recreational activity. Determinants: qualifications, years of experience, and pain education. Nurses had significantly more skills and knowledge to assess and alleviate pain. Care workers disagreed more with: not complaining means there is no pain, and that older people have lower pain sensation. | Increases in education about pain, and strengthening cooperation between nurses and care workers to manage pain, in order to provide effective and active chronic pain care, are recommended. |
| 96 | Taylor (2014)  UK | 203 carers in 54 residential homes | Evaluation of training; to evaluate training sessions (DISDAT and pain management) | DISDAT; pain management training for managers and carers of residents with learning disability | Opinions about the training in pain assessment | DISDAT facilitates pain assessment. Carers’ pain assessment and management skills improved and facilitated more individualized interventions. Important to train non-registered staff. Most useful elements: information on pain medication and tools. | Pain assessment tools for people with communication/ learning disabilities are needed. Training for non-registered staff also recommended. |
| 97 | Torvik (2015) Norway | 392 nurses, mean working experience 15 years | Cross-sectional; To explore pain assessment tools in home care and NHs | No pain assessment tool; no intervention | Use of tools and determinants; pain treatment self-reported competence | Pain assessment tools were not commonly used in either of the settings. Regular training in the use of these tools was related with using them more often. | Important to regularly train staff in the use of pain assessment tools. |
| 98 | Torvik  (2010) Norway | 77 severely cognitive impaired residents; mean age 86 SD 6.6; 75% female | Cross-sectional; To examine Doloplus-2 use, validity and reliability | Doloplus-2; no intervention | Reliability and validity; comparison of pain scores | Staff evaluated significantly more patients with pain when using the scale, than with proxy-rated pain. For 29% of the residents, the registered nurses couldn’t identify pain. | Doloplus-2 is a useful tool for estimating pain in nonverbal residents. Nurses should use the tool and their clinical experience together. |
| 99 | Tousignant-Laflamme (2012)  Canada | 72 staff; mean age 41.4 SD 8.8; 91.6% female; median of 10 years of practice in NHs. | Qualitative study; To identify the pain educational needs of health care providers | No pain assessment tool; no intervention | Topics to be addressed in a pain education program | Main needs: pain assessment; pharmacological management (drug interactions, adverse effects, substitution of drugs); non-pharmacological modalities; pain neurophysiology; clinical signs of pain; and staff communication strategies. | Even though staff have experience (>10 years), a comprehensive pain management educational program is needed. |
| 100 | Trinkoff (2015)  USA | 1142 nursing homes | Secondary data analysis; to examine association of administrator and director education/certification with resident outcomes | No pain assessment tool; no intervention | Residents outcomes | Nursing homes led by administrators with a master degree or higher, and certification had significantly better pain outcomes. Same when NH led by director of nursing with a bachelor degree or higher plus certification. | Quality of resident care can improve with in-service and professional development education leading to certification for administrators and directors of nursing. |
| 101 | Tse  (2018)  Hong Kong | 53 residents from 4 NHs; 71% female; mean age 83.3 SD 7.5 | Cluster-RCT; To explore the effectiveness of a play activities program among residents with dementia | PAINAD; 1-hour play activities program (8 weeks) versus reading books/magazines for 15 minutes | Pain, depression, happiness, ADL, social engagement, behavioural symptoms, mobility | Play activities had a significant treatment effect on pain, its intensity, and satisfaction with non-pharmacological therapy when compared to control group. | Inclusion of play activities in usual care might be beneficial for residents and their caregivers. |
| 102 | Tse  (2013)  Hong Kong | 90 residents. Intervention (n=48) 72.9% female. 33 staff in intervention (all female, age range 36-45) | Quasi-experimental; to enhance pain management via a program for staff and residents | Geriatric Pain Assessment; Integrated pain management program (8 wks) (staff education and activities for residents) | Knowledge and attitudes; pain; type of therapies; psychologic well-being indicators | Intervention group improved knowledge and attitudes towards pain management. Staff felt more confident and had a more positive attitude in pain management and in using non-drug therapies. Residents significantly decreased pain, improved well-being, and used more nondrug strategies. | A focus on holistic approaches to pain management, given that it provides benefits to both staff and residents, is recommended. |
| 103 | Tse  (2016)  Hong Kong | Total 50 residents (60-99 years; 82% female) Intervention group (n=32) | Quasi-experimental (12 weeks); To examine the feasibility of a peer-led pain management program | Numeric Rating Scale; Intervention (group-based peer-led pain management program, training, demonstration of nondrug strategies) vs control group (1 session of pain management per week) | Pain, participants´ self-efficacy in pain management, performance in daily living activities, loneliness, subjective happiness | Significant reduction in pain intensity and improvement in activities of the daily living in both groups. Intervention group significantly increased happiness. Loneliness dropped significantly in both groups. The volunteers had a significant increase in pain management knowledge, and self-efficacy. | Peer-led pain management program was feasible and can relieve chronic pain in residents. |
| 104 | Vaismoradi (2016)  Norway | 6 included studies | Meta-synthesis; to understand older people’s pain experiences of and perspectives of pain management | No pain assessment tool; no intervention | Perspectives and experiences on pain and pain management | Residents thought: pain assessment is difficult, staff needs a method for pain validation, and to take it seriously. When pain is not identified, they feel unsafe. Barriers to pain management: not trusting methods for pain assessment, residents don’t want to be seen as complainers. When residents consider pain avoidable, they are more likely to use non-drug therapies. When they take pain as part of ageing, they can have fear towards medication. | Residents need to be encouraged to report their pain. Healthcare staff require training to take a person-centred approach towards pain practice. |
| 105 | Van Herk (2009)  Netherlands | 174 residents (median age 82; 63.2% female); 293 proxies (caregivers/  relatives) | Cross-sectional; To compare pain reports of NH residents with ratings by proxies | Numeric Rating Scales; no intervention | Agreements on ratings; proxies’ certainty about their observations | Residents judged pain intensity at rest significantly higher than proxies. Caregivers scored significantly higher pain ratings for residents on analgesics. Proxies´ reports on pain and its intensity is unreliable, especially for cognitively impaired persons. | Education of caregivers about chronic pain assessment and treatment could improve pain management. Relatives should learn how to treat pain through non-pharmacological interventions. |
| 106 | Veal  (2019) Australia | 477 residents; median age was 85.1; 65.3% female | Retrospective study; To measure the prevalence and assess pain management | Tool not mentioned; no intervention | Prevalence of pain; pain management | Pain was usually treated by analgesics (45.5%), massage (40.7%) and heat packs (13.8%). Residents with dementia were less likely to have pain identified on their most recent pain assessment. | GPs should consider the suitability of using “as required” analgesics. GPs should ensure medical records contain information about pain and its potential causes, so staff are vigilant. |
| 107 | Veal  (2018) Australia | 23 staff (nurses/managers); median aged care experience 20 years | Qualitative study; to characterize pain management and identify barriers | No pain assessment tool; no intervention | Barriers to optimal pain management | Pain was informally assessed. Barriers: difficulty identifying and assessing pain, resident’s resistance to report it and/or taking medications, and staff communication barriers (nurses- GPs). To improve: consistent documentation, staff communication, and pain education for staff, residents and relatives. | Education about pain management should be given to residents, family, nurses and carers. |
| 108 | Zwakhalen (2018) Netherlands | 810 Health care professionals and care assistants | Cross-sectional; To explore use of pain assessment tools and guidelines in Europe | No pain assessment tool; no intervention | Use of pain assessment tools | International/local pain standards/tools are not commonly used. 58% don’t use any tool. 34% use observational tool for residents with dementia. Nurses recognize a lack of education regarding pain. Barriers to use behavioural pain assessment tools: uncertainty about observation; lack of information, objectivity, education, knowledge, expertise, time, interest and awareness. | Pain education is needed in all countries. When designing tools and guidelines it is important to consider settings and types of pain. It’s necessary to improve clinical usefulness of tools, and increase user’s confidence in their reliability and validity. |
| 109 | Zwakhalen (2012) Netherlands | 22 residents; mean age 80 SD 8.6; 68.18% female; most with severe cognitive deficit; 6 nursing staff | Descriptive study; To investigate the feasibility of regular pain assessment using an observational scale residents with dementia, and determine interventions. | PACSLAC; Regular pain assessment using an observational scale | Pain status, feasibility and experiences of staff when conducting regular pain assessment | Using observational scale twice per week had a good compliance. Described as user friendly and feasible. However, staff would prefer to use it once per week. Some had difficulties when interpreting pain cues. In 35% of the cases when pain score indicated treatment, this didn’t happened. Non-pharmacological interventions (particularly comforting and distraction) were mainly used. | Providing pain assessment tools to nursing staff is not enough to change pain management practices. |

*QOL: Quality of life. CAN: certified nursing assistant. PACSLAC- Pain Assessment Checklist for seniors with Limited Ability to Communicate. PAINAD - Pain Assessment in Advanced Dementia. RCT: Randomized Controlled Trial. NHs – Nursing Homes. NAs/ANs – nursing assistants. RNs – registered nurses. LTC: Long term care. ADL- Activities of Daily Life. NRS- Numerical Rate Scale. NOPPAIN: Non-communicative Patient’s Pain Assessment instrument scale. PRN – *pro re nata*, medication as needed. MDS-RAI - Minimum Dataset of the Resident Assessment Instrument pain scale. LTCF: Long term care facility. DISDAT: Disability Distress Assessment Tool. SF-36: Short Form-36 Health Survey.
